# Computation-aided design of rod-shaped nanoparticles for tumoral targeting

**DOI:** 10.1016/j.jconrel.2025.114169

**Published:** 2025-08-26

**Authors:** Jinhyung Lee, Wuxia Zhang, Danh Nguyen, Libo Zhou, Justin Amengual, Jin Zhai, Trystin Cote, Maxwell Landolina, Elham Ahmadi, Ian Sands, Neha Mishra, Hongchuan Yu, Mu-Ping Nieh, Kepeng Wang, Ying Li, Yupeng Chen

**Affiliations:** a Department of Biomedical Engineering, University of Connecticut, Storrs, CT 06269, United States of America; b Department of Mechanical Engineering, University of Wisconsin-Madison, Madison, WI 53706, United States of America; c Department of Immunology, University of Connecticut Health Center, Farmington, CT 06030, United States of America; d Department of Pathobiology and Veterinary Science, University of Connecticut Health Center, Farmington, CT 06030, United States of America; e Polymer Program, Institute of Materials Science, University of Connecticut, Storrs, CT 06269, United States of America; f Department of Chemical and Biomolecular Engineering, University of Connecticut, Storrs, CT 06269, United States of America; g The Warren Alpert Medical School of Brown University and Rhode Island Hospital, Providence, RI 02903, United States of America

**Keywords:** Computation-aided design, Nanomaterials, Drug delivery, Rod-shaped nanoparticles

## Abstract

Various nanomaterials have been developed for drug delivery, but the vast majority are spherical nanoparticles (50–500 nm in diameter). This limits their ability to target and infiltrate hard-to-penetrate tissues, such as certain solid tumors with a dense extracellular matrix (ECM). To investigate how the key physical parameter of shape influences tumor targeting, we developed Janus base nanoparticles (JBNps), a family of rod-shaped delivery vehicles that self-assemble into nanotube bundles with encapsulated drug cargos. In a head-to-head comparison with spherical nanoparticles, the rod-shaped JBNps enabled significantly better tumor infiltration and targeting (by 300 %) of an ECM-rich adenocarcinoma model. To efficiently optimize formulations, we employed a computation-aided methodology to refine drug loading and design parameters, which achieved maximal drug loading efficiency (by 97 %). This work provides a broadly applicable strategy for designing nanoparticles and highlights the potential of exploiting the physical shape of nanoparticles to achieve enhanced penetration and targeting of solid tumors.

## 1. Introduction

Significant challenges remain in drug delivery, particularly in targeting hard-to-reach tissues such as solid tumors, which have a dense and complex extracellular matrix (ECM) [1]. Although various drug delivery materials have been developed, the majority involve spherical nanoparticles with diameters ranging from 50 to 500 nm, which face significant physical constraints in effectively penetrating tissues with a dense ECM and small pores [2,3]. While considerable efforts have been devoted to optimizing the chemical properties of nanoparticles, there has been only limited research on how their shape influences drug delivery. Nonetheless, some early studies suggested that more rod-shaped nanoparticles can overcome the limitations of spherical nanoparticles and improve tissue penetration [4,5]. However, there is a lack of comprehensive, head-to-head comparisons showcasing the benefits of rod-shaped nanoparticles in specific applications. The use of this approach has also been impeded by the fact that current rod-shaped nanoparticles (such as carbon nanotubes and gold nanorods) are not biodegradable and are not versatile in terms of the range of drug cargo that they can carry [6–8] .

Herein, we introduce a novel family of rod-shaped nanoparticles derived from DNA-inspired Janus base nanomaterials (JBNs). These nanoparticles are composed of DNA-mimicking bases, specifically guanine and cytosine, conjugated with an amino acid side chain. The unique chemistry, structure, and formation mechanisms of JBNs set them apart from conventional nanomaterials, including lipids, polymers, carbon nanotubes (CNTs), and DNA origami structures. We previously demonstrated that a JBN-based delivery platform (JBNp) could deliver small RNAs into cell *via* endosomal escape more effectively than conventional lipid nanoparticles (LNPs) could and was more biocompatible than CNTs and cationic polymers [9]. These effects can be attributed to the unique biomimetic structure of JBNps, which results in reduced toxicity and immunogenicity. Moreover, the JBNps are highly versatile in the range of drugs that they can deliver, being applicable for the intracellular delivery of both small chemical molecules and large biological molecules, such as siRNA and mRNA.

In this study, we used a computation-aided design approach to develop rod-shaped Janus base nanoparticles (Rod JBNps) capable of delivering a wide range of cargo, including small molecules and RNAs. Conventional methods for nanoparticle formulation have often relied on a resource-intensive and time-consuming “Edisonian” approach, which is predominantly characterized by trial and error and lacks a systematic and theoretical framework [10,11]. Recent advances in high-fidelity computation-aided design have provided deep insights into the properties and behaviors of nanomaterials, leading to nanoparticle-based drug delivery systems that are more effective and efficient [12,13]. In this paper, we present a streamlined method using computation-aided design to optimize JBNp formulations, in terms of their surface charge, pH, and aging time, to maximize drug loading and delivery. The methodology is theoretically conceptualized and experimentally validated using small-angle X-ray scattering (SAXS), along with a comprehensive *in vitro* and *in vivo* evaluations of the ability of JBNps to deliver cargo. Importantly, we also identified that rod-shaped JBNps exhibit superior penetration into ECM-rich adenocarcinoma tumors, achieving significantly deeper infiltration into tumor organoids and solid tumors compared with non-rod shaped JBNps and the FDA-approved spherical nanoparticle, Doxosome. We also applied JBNps to a small animal model as a proof-of-concept and showed that they dramatically enhanced tumor targeting (~300 %) and improved treatment efficacy compared with Doxosome. These findings highlight the potential of exploiting the physical shape of Rod JBNps to achieve enhanced penetration and targeting of solid tumors. Unlike current antibody-drug conjugate (ADC) strategies, this targeting mechanism relies on physical factors rather than specific tumor antigens [14,15]. Consequently, this approach can overcome the limitations associated with the specificity of tumor surface markers and the heterogeneity of tumor cell expression profiles, offering broader applicability for the targeting of solid tumors. In summary, this study integrates nanoparticle engineering with computation-aided methodology, not only providing a new design strategy for developing shape-based nanoparticle but also demonstrating how computation-aided methods can optimize nanoparticle formulations.

## 2. Results

### 2.1. Simulation and experimental validation of JBNt drug loading

**Fig. 1.**
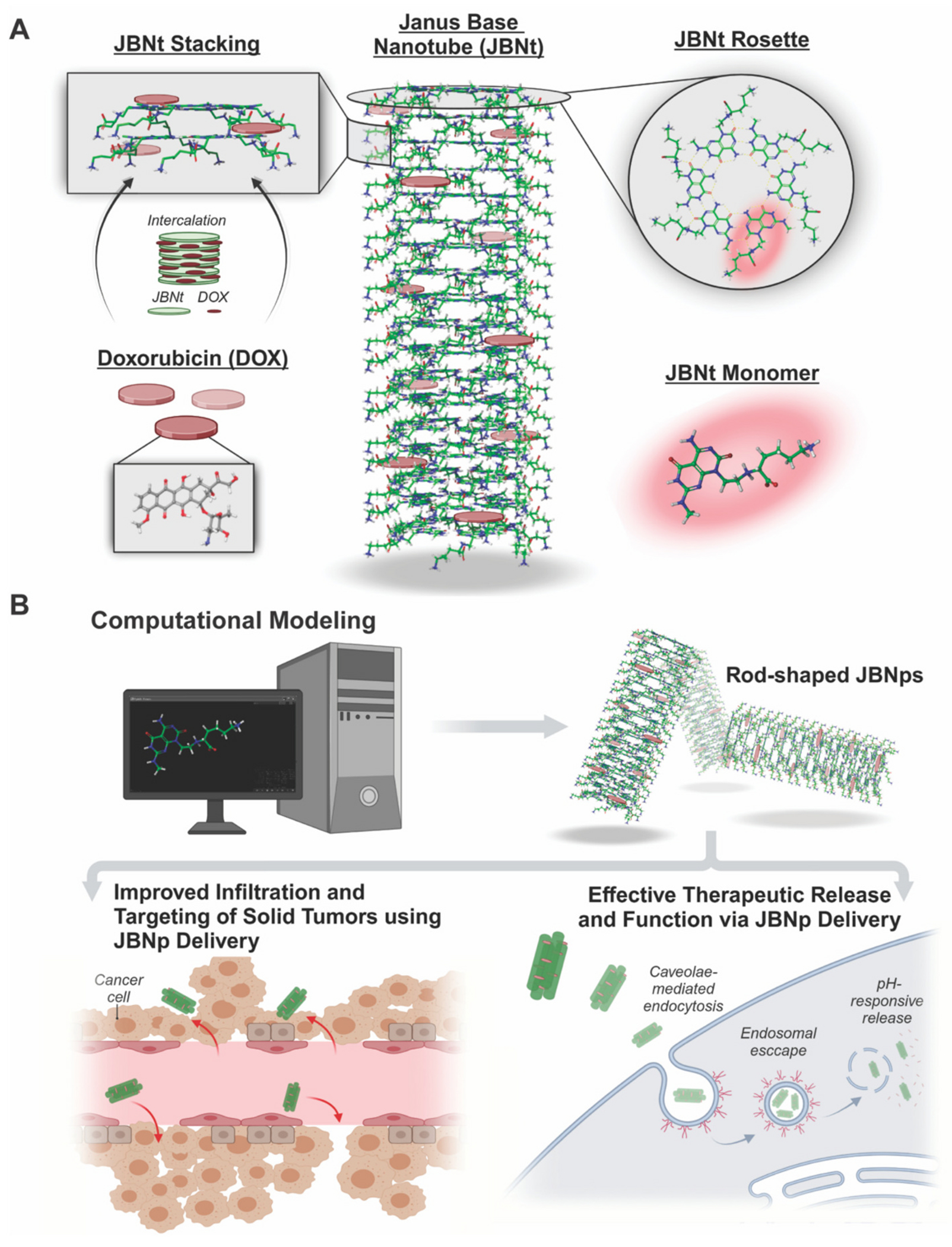
Schematic illustration of the Janus Base Nanoparticles (JBNps) for drug delivery. (A) Schematic illustration of self-assembly of the Janus base Nanotube (JBNt) and its components. (B) The scheme shows the utilization of computation-aided predictive modeling to assemble JBNt-cargoes and engineer rod-shaped nanoparticles called Rod JBNps. As a proof-of-concept, Rod JBNps infiltrate the ECM and release cargoes in “hard-to-penetrate” tissue such as a solid tumor.

**Fig. 2.**
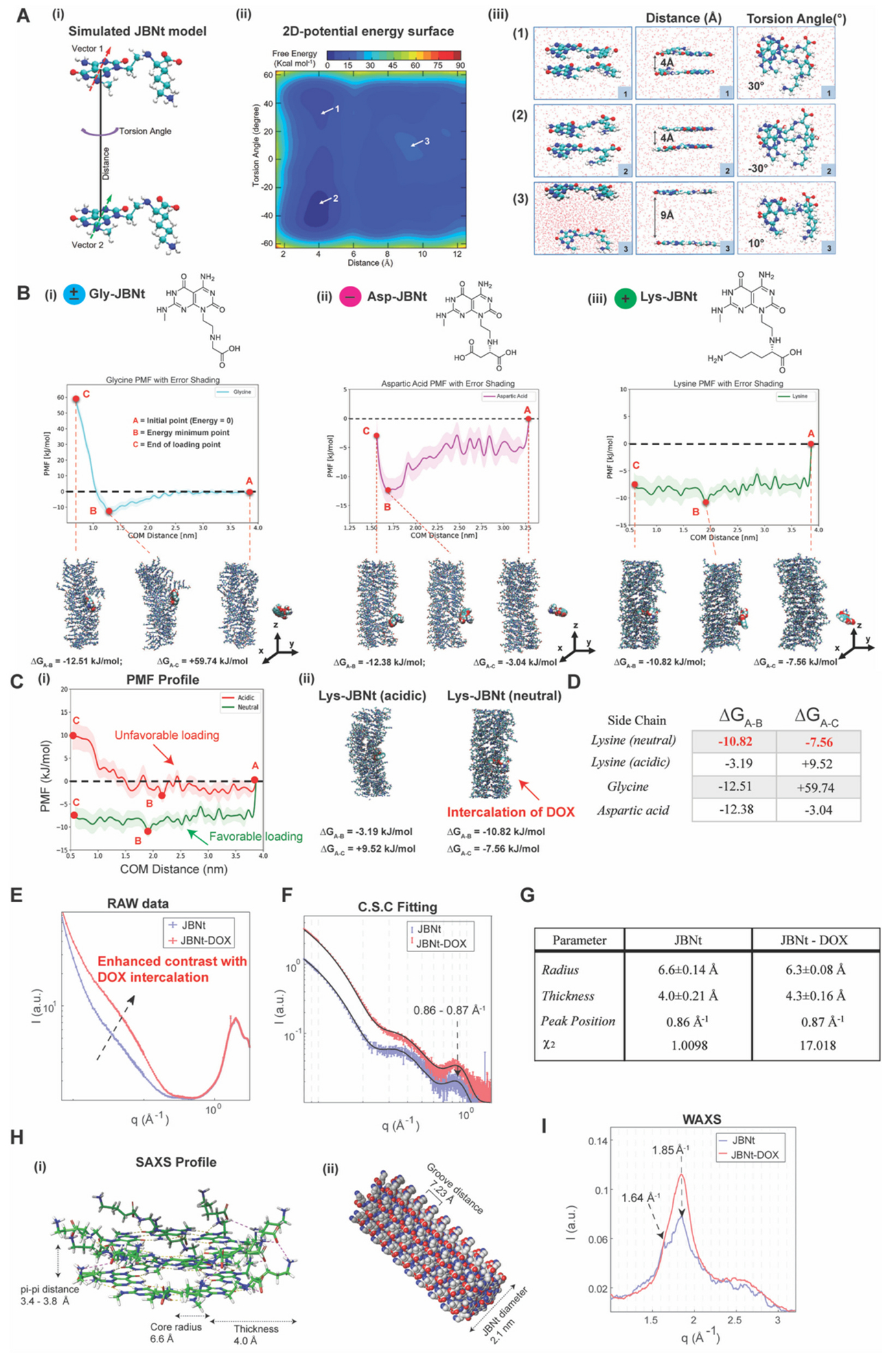
Simulation and Experimental Validation of JBNt’s drug loading. (A, i) Metadynamics simulation of stacking two G^C Janus base monomers considering the distance and torsional angle between two monomers. (A, ii) 2D potential energy surface of the stacking interaction between two monomers. (A, iii) Representative snapshots of stacking of two monomers. (B, i) Potential of mean force (PMF) of DOX loading onto Glycine-JBNt (Gly-JBNt). (B,ii) PMF of DOX loading onto Aspartic acid-JBNt (Asp-JBNt). (B,iii) PMF of DOX loading onto Lysine-JBNt (Lys-JBNt). (C,i) PMF of DOX loading onto Lys-JBNt at neutral pH and acidic pH. (C, ii) Representative snapshots of the intercalation of DOX at different pH values. (D) Table of PMF profiles of DOX loading onto different JBNts; “A” represents initial point (energy = 0), “B” represents energy minimum point, “C” represents end of loading point. (E) Enhanced intensity due to DOX intercalation at identical Lys-JBNt concentrations. (F) Small-angle X-ray scattering (SAXS) data and their best fits using the core-shell cylinder and Gaussian peak combined model. (G) Tabulated best-fitting parameters for the SAXS data. (H) Detailed local structure of Lys-JBNt (I) Two high-q peaks corresponding to the well-defined interlayer spacings of Lys-JBNt monomers.

Janus base nanotubes (JBNts) are self-assembled into rosette structures from a library of nanotube monomers that consist of two components: a base component mimicking DNA bases and an amino acid side chain. The cargo (doxorubicin in this case) is encapsulated within the JBNt through intercalation between JBNt monomers (Fig. 1A and Fig. 1B). Two key geometrical parameters—namely, the vertical distance and the relative torsion angle between two G^C Janus base monomers—can be varied to investigate JBNt self-assembly (Fig. 2A, i). Fig. 2A, ii shows 2D-potential energy surfaces of relative stacking energies between two G^C Janus base monomers as a function of these two variables. Fig. 2A, iii displays the stacked G^C Janus base monomers’ representative configurations, locally optimal vertical distances, and relative torsion angles. The relative stacking energy of G^C Janus base monomers concerning the vertical distance and relative torsion angle reveals one optimal stacking minimum at approximately 4 Å and 30^°^, respectively. This geometry facilitates π–π stacking for structural stability [16] and provides an optimal intercalation gap for drug loading [17,18]. We then modeled the intercalation of doxorubicin (DOX) into JBNts. We selected three JBNt candidates from our library—namely, Gly-JBNt, Asp-JBNt, and Lys-JBNt—with three different surface charges of the side chain: glycine (neutral), aspartic acid (negative), and lysine (positive), respectively. We then calculated the potential mean force (PMF) of each JBNt involved in the intercalation of DOX (Fig. 2B i–iii). Among the JBNt candidates, Lys-JBNt displays the lowest Δ*G_A-B_* = −10.82 kJ/mol, and Δ*G_A-C_* = −7.56 kJ/mol, indicating the relatively lower energy barriers for both DOX absorption into the JBNt side chain and for the intercalation of DOX within JBNt compared with those for Gly-JBNt and Asp-JBNt. Lys-JBNt was thus selected for DOX loading. We further investigated the capacity of the DOX molecule to bind to JBNt under various pH, including neutral and acidic. Fig. 2C, i illustrates the PMF profiles of Lys-JBNt and DOX molecules in the water phase at different pH. The calculated free energy of the system gradually decreases as DOX approaches the center of the JBNt, indicating a favorable DOX intercalation process in the neutral environment. Conversely, DOX encounters challenges when entering the center of the JBNts under low-pH conditions. We then calculated the DOX loading energy under different pH from the PMF profiles. By subtracting the final loading energy from the initial loading energy, we determined binding energy of −7.56 kJ/mol and 9.52 kJ/mol for DOX to Lys-JBNts under neutral and acidic conditions, respectively (Fig. 2C, ii). These values confirm that DOX is more favorably loaded onto Lys-JBNts under neutral conditions. All the energy values associated with the loading of DOX onto different JBNts are summarized in Fig. 2D.

The 1D small-angle-X-ray scattering (SAXS) data (after transmission and water background correction) with reduced to one dimensional intensity profile were analyzed and best fitted *via* SasView software using a model that combines a core-shell cylinder (CSC) morphology with a single Gaussian peak. Fig. 2E indicates that the intensity of scattered X-ray was enhanced after the loading of DOX. The best fit using this combined model agrees well with the SAXS data. The validation of the CSC model suggests that the Lys-JBNt selected from the previous simulations forms a hollow rod structure, consistent with the proposed structure (Fig. 2F). The shell of the cylinder represents the Lys-JBNt. The best fitting results (Fig. 2G) indicate a consistent JBNt diameter of ~2.1 ± 0.24 nm, suggesting that DOX loading does not markedly alter the JBNt radial dimension. Notably, electron densities of the core (ρ_c_), shell (ρ_s_), and water (ρ_w_) are in the order of ρ_s_ > ρ_c_ > ρ_w_, which is consistent with the calculated electron densities. The best-fitted scattering contrast (the difference in electron density) between core and water decreases after DOX loading, while that between the shell and water increases. This indicates that DOX (with a higher electron density) is associated with the JBNt shell, resulting in a higher electron density. A small proportion of DOX also localize near the core, thus increasing ρ_c_ and reducing the difference between ρ_c_ and ρ_w_. The increased intensity at equivalent JBNt concentration results from enhanced scattering contrast between the CSC and solvent, consistent with DOX intercalation with the JBNt.

Further investigation of the JBNt rosette structure reveals that the best-fitted Gaussian peak position, located at 0.87 Å^−1^, corresponds to a regular repeat spacing of 7.2 Å. The spacing can be attributed to the detailed local structure of the JBNt (Fig. 2H). Wide-angle-X-ray scattering (WAXS) analysis of two samples, examined in the presence and absence of DOX (Fig. 2I), reveals two distinct peaks at 1.64 Å^−1^ and 1.85 Å^−1^, corresponding to spacings of 3.83 Å and 3.40 Å, respectively. These spacings represent the two distinct JBNt groove distances observed after the full rotation of JBNt rosettes (Fig. S6). Notably, the sum of these two spacings is 7.23 Å, which closely aligns with the Gaussian peak spacing of 7.2 Å. This suggests that spacings of 3.83 Å and 3.40 Å likely alternate within the JBNt rosette structure. These findings suggest that we have successfully conducted a simulation of JBNt to determine the optimal side chain and condition to intercalate cargo, confirming our findings through SAXS analysis.

### 2.2. Response surface methodology-based computational method to optimize JBNp

**Fig. 3.**
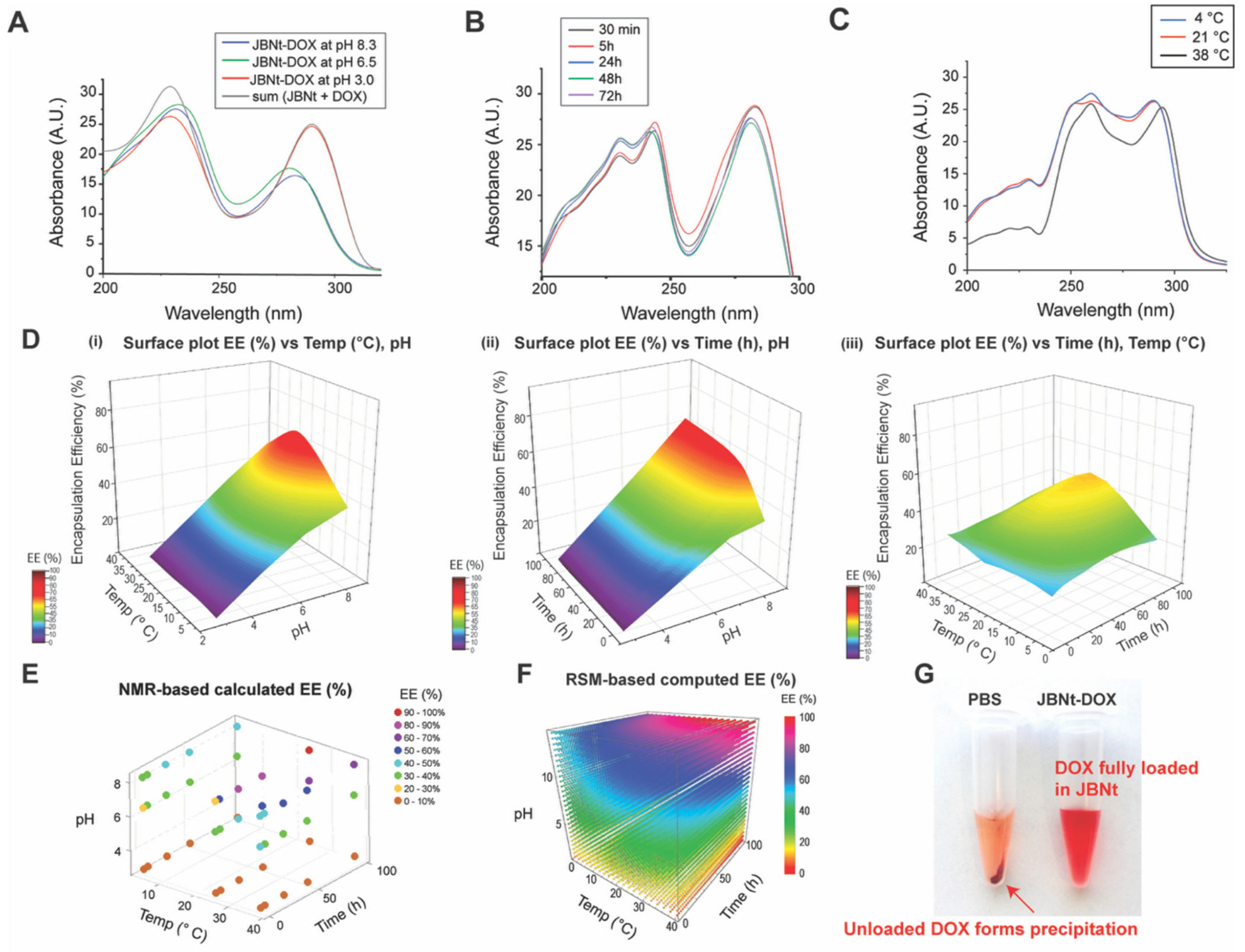
Optimization of JBNp formulation based on RSM-computed method. (A) UV–Vis analysis of pH-dependent assembly after 72 h at 38° C. (B) UV–Vis analysis of time-dependent assembly at 38° C and pH 8.3. (C) UV–Vis analysis of temperature-dependent assembly after 72 h at pH 8.3. (D) Response surface methodology of DOX loading to the JBNt. 2D RSM contour plots for the EE(%) with different conditions; (i) temp, pH. (ii) time, pH. and (iii) time, temp. (E) NMR-based calculated encapsulation efficiency EE (%). (F) Three-dimensional cube figure of RSM-based computed EE (%). (G) Photograph of solution of DOX (left) and JBNt intercalated DOX (right) in the ambient light.

To validate the simulation, the UV–visible (Vis) spectra were obtained to demonstrate the molecular-level incorporation between JBNts and DOX. Upon assembly with DOX, the intensity of the 280-nm peak of JBNt-DOX decreased due to the intercalation between JBNt units and DOX. After 72 h at pH 8.3 and 38 ^◦^C, a significant decrease in absorbance at 280 nm was observed compared with that in the controls, demonstrating the successful loading of DOX into JBNts under the explored simulated conditions (Fig. 3A–C). Next, we assessed the encapsulation efficiency (EE) across various pH through nuclear magnetic resonance (NMR) spectroscopy (Fig. S5A). Fig. 3E shows the NMR-based calculated EE with the variation of three variables (pH, temperature, and time). The results correspond to the trends shown in the UV–Vis spectra. To optimize the JBNt-DOX further, we employed a computational approach based on the response surface methodology (RSM). Fig. 3D (i, ii, iii) presents the three-dimensional RSM surface plots, demonstrating the incorporation of DOX into JBNt under variation in the variables of temperature, pH, and time. The three abovementioned factors and their relationships to the EE were together incorporated into a threedimensional cube computation, as shown in Fig. 3F. Significant relationships between factors and the response were observed *via* ANOVA. pH and time showcased high principal effects on EE. Temperature, on the other hand, maintained a low principal effect but high square effect. These findings indicate that pH and time possess linear relationships with EE, while temperature’s relationship is quadratic. The optimal temperature was found to be centered in the tested values, as high or low temperatures resulted in poorer EE. However, as pH and time increased in a linear fashion, the EE simultaneously increased similarly. After analyzing these results, we performed a confirmation study using the conditions theorized to be optimal. We successfully maximized the EE to 93 % of DOX being loaded into JBNt, within the higher end of the 95 % prediction interval (0.5348, 0.9628) observed in the model. This optimal JBNp showed a significant decrease in UV–Vis absorbance and a hypsochromic shift (blue shift), indicating successful drug intercalation into the JBNt structure (Fig. S5B). The observed blue shift suggest strong *π*- *π* stacking interaction between the aromatic rings of DOX and the *π*-conjugated system of the JBNt backbone, which indicates higher energy transition, leading to a shift toward shorter wavelength. We also found that the JBNt-DOX solution changed to a saturated red colour in PBS (Fig. 3G). As another example of the ability to incorporate cargo into JBNt, we also effectively loaded resveratrol, a promising antioxidant and anticancer drug (Fig. S7).

### 2.3. Fabrication and characterization of rod-shaped JBNps

**Fig. 4.**
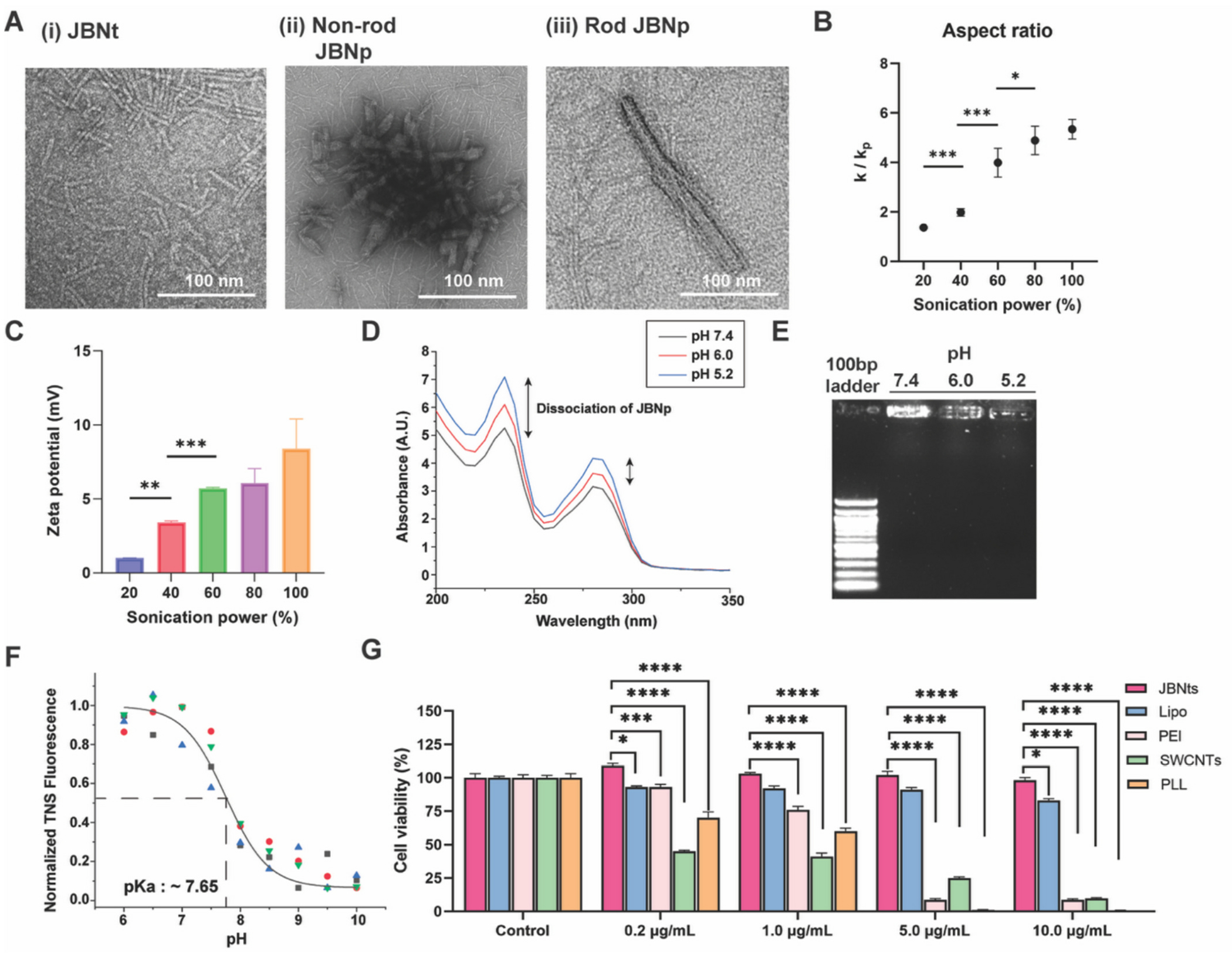
Fabrication and characterization of rod-shaped JBNps. (A) Transmission electron microscope (TEM) images of JBNts, Non-rod JBNp, and Rod JBNp. (B) Aspect ratio analysis. k/k_p_ represents length/width. (C) Zeta potential analysis varying sonication power. *n* = 3. (D) UV–Vis analysis. (E) Agarose gel electrophoresis assay varying pH. (F) Apparent pKa assay. n = 3. (G) *In vitro* cytotoxicity assay: Lipofectamine2000 (Lipo), single-walled carbon nanotubes (SWCNTs), polyethyleneimine (PEI), and poly-l-lysine (PLL). The data were expressed as the percentage of surviving cells; the values are mean ± SEM (*n* ≥ 10). *P < 0.05, ** *P* < 0.01, and *** *P* < 0.001 compared to untreated control.

Guided by MD simulations, we can further process JBNt with DOX molecules into delivery vehicles. Transmission electron microscope (TEM) was used to evaluate the rod-shaped morphology of the fab- riacated delivery vehicle. JBNt alone presents nanotubular morphology (length of ~100 nm) **(**Fig. 4A, i and Fig. S10A), capable of forming nonrod-shaped bundles of nanotubes after its incorporation of with cargoes (Fig. 4A, ii). Interestingly, a simple sonication process can break these bundles into smaller individual rod-like vehicle (named Rod JBNp, Fig. 4A, iii). Although the overarching JBNp architecture is formed by noncovalent interactions of their small molecule units and cargo, JBNps alone are stable entities, as evidenced by the unchanged UV–Vis absorbance intensity of Rod JBNps over a 14-day period (Fig. S8). As shown in Fig. 4B and Fig. S10B, we altered the sonication power to process Non-rod JBNp to Rod JBNp. The findings showed that their aspect ratio (AR), the length (k) divided by the width (*kp*), increases as sonication power (%) increases. The Rod JBNp’s AR was 5.3, which has a length of 126.3 ±13.9 nm, and a width of 26.7 ± 2.5 nm. The zeta potential measurements of the JBNps demonstrated a shift of their surface charge varied the sonication power (Fig. 4C). For subsequent analysis, we fixed the sonication power to 100 % and altered the sonication time. As shown in Fig. S10C and Fig. S10D, a sonication time of 150 s resulted in the formation of Rod JBNp with a surface charge of ~7 mV. UV–Vis spectra demonstrated a dissociation between JBNps and DOX upon decreasing the pH from 7.4 to 5.2 (Fig. 4D). Moreover, as shown in Fig. 4E, agarose gel electrophoresis was used to assess the release mechanism of loaded cargo under acidic condition. The result demonstrated that Rod JBNp dissociated into their component parts upon decreasing the pH to 5.2, resulting in the release of cargoes. The apparent pKa of Rod JBNp was determined to be ~7.65 by the 2-(p- toluidine)-6-naphthalene sulfonic acid (TNS) binding assay, which is in the optimal range for tumor delivery and escapes from the endosome *via *the “proton sponge” effect (Fig. 4F). The amine groups in JBNts bind protons in the endosome (“proton sponge” effect), introducing positive charges to Rod JBNps and facilitating the release of small molecule drugs through charge repulsion. We also evaluated the cytotoxicity of the JBNps. Compared with various commonly used delivery materials, Lipo, SWCNTs, PEI, and PLL, the JBNps showed significantly better cell viability (Fig. 4G), with relative cell viabilities of fibroblast cells following incubation with delivery materials of increasing concentrations.

### 2.4. In vitro delivery and functional assay of rod JBNps in cancer cells and spheroids

**Fig. 5.**
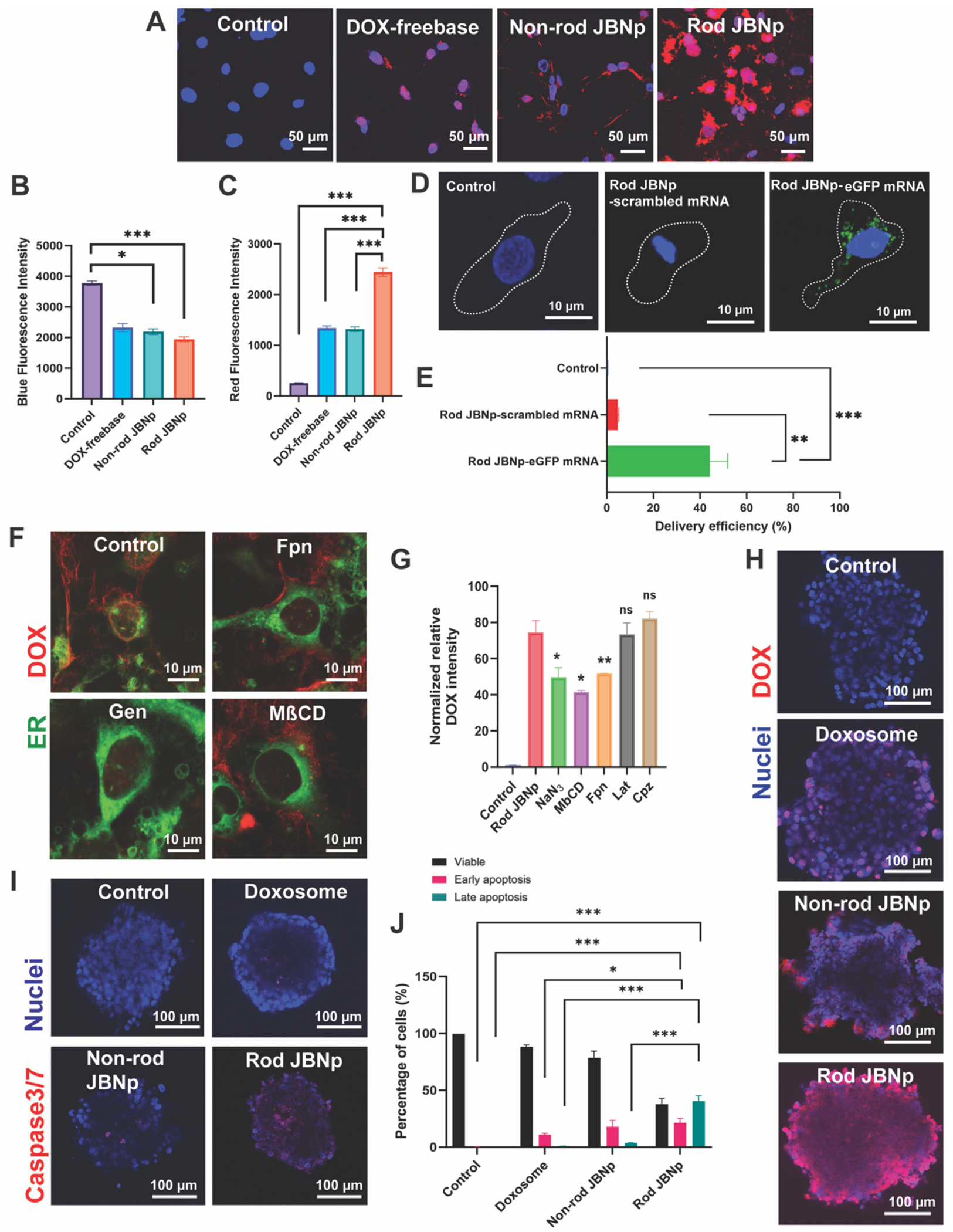
Rod-shaped JBNps showed efficient delivery to cells and cancer spheroids. (A) CLSM images of DOX delivered by the Rod JBNps into SKOV3 cells; nuclei (blue); DOX (red). (B) Statistical analysis of mean DOX (red) fluorescence intensity. (C) Statistical analysis of mean nuclei (blue) fluorescence intensity. (D) CLSM images of eGFP encoded mRNA delivered by Rod JBNps into SKOV3 cells. (E) Flow cytometry analysis delivering eGFP encoded mRNA via Rod JBNps. (F) CLSM images of Rod JBNp pretreated with caveolae-mediator inhibitors of Genistein (Gen), methyl-*β*-cyclodextrin (M*β*CD), a fillipin (Fpn]; Endoplasmic reticulum (green); DOX (red). (G) Quantitative analysis of Rod JBNp uptake. (H) CLSM images of SKOV spheroids treated with indicated group and stained with Hoechst (blue) and Caspase 3/7 (red) reagents. (I) CLSM images of SKOV3 spheroids delivered by the Rod JBNp; cell nuclei stained with DAPI (blue); DOX (red). (J) Apoptosis assay of SKOV3 spheroids after treatments for 48 h using Apoptosis Kit with Annexin V FITC and PI. Percentage of viable, early, and late apoptotic cells. Data are presented as the means ± SEM of triplicate experiments. *P < 0.05, **P < 0.01, and ***P < 0.001. (For interpretation of the references to colour in this figure legend, the reader is referred to the web version of this article.)

Based on their excellent drug loading capacity, Rod JBNps have great potential for use as vehicles for intracellular drug delivery. To demonstrate the ability of Rod JBNps to deliver cargo into cells, we loaded small molecule as DOX to be delivered to the ovarian cancer cell (SKOV3). As shown in Fig. 5A imaged by confocal laser scanning microscope (CLSM), Rod JBNp efficiently delivers DOX as DOX (red) fluorescence was observed in the nucleus (blue). We then compared the delivery efficiency of DOX between Non-rod JBNp and Rod JBNp. As presented in Fig. 5B, the mean DOX intensity of the Rod JBNp was significantly higher than that of treated with the Non-rod JBNp and DOX-freebase groups, indicating that DOX was delivered into the nucleus more effectively by Rod JBNp compared than in controls. The mean fluorescence intensity of DAPI for each group was also statistically analyzed. As indicated in Fig. 5C**,** the fluorescent intensity of DAPI in the Rod JBNps group was significantly weaker than that of controls, which may have been caused by the failure of DAPI to bind with the damaged DNA in Rod JBNp treated cells. The results showed that the Rod JBNps delivered DOX into cells in a time-dependent fashion, showing a red signal (DOX) in the cytoplasm (Fig. S11). To demonstrate the capability of Rod JBNps to deliver various cargo types into cells, we also loaded messenger RNA (mRNA) encoding enhanced green fluorescent protein (eGFP) into the Rod JBNps and delivered it to SKOV3 cells, resulting in successful translation and expression of eGFP in the cytoplasm (Fig. 5D). Furthermore, the efficiency of Rod JBNp-eGFP mRNA (Rod JBNp loaded with eGFP mRNA) delivery was quantified by flow cytometry, demonstrating significantly higher delivery efficiency compared to negative control, Rod JBNp-scrambled mRNA (non-targeting mRNA) (Fig. 5E). In addition, Rod JBNps can co-deliver siRNAs along with DOX to SKOV3 cells (Fig. S12). This demonstrates the versatility of Rod JBNps in delivering a diverse range of cargoes. Moreover, because the JBNps have a chemical composition that differs from those of conventional delivery materials, we also explored the mechanism behind the cellular uptake of Rod JBNps. We pretreated SKOV3 cells with several endocytic inhibitors, including an ATP inhibitor (NaN_3_), caveolae inhibitor (Gen- istein [Gen], methyl-β-cyclodextrin [*MβCD*], a fillipin [Fpn]), a clathrin inhibitor (chlorpromazine [Cpz]), and a macropinocytosis inhibitor (latrunculin A [Lat]). As shown in Fig. 5F**,** qualitative imaging demonstrated significant inhibition of uptake and delivery to the endoplasmic reticulum (ER) using caveolae-mediated inhibitors. Similarly, the quantitative results presented in Fig. 5G further demonstrated substantial uptake inhibition of uptake upon the use of caveolae- mediated inhibitor. Thus, the results suggest that the uptake mechanism of Rod JBNps–DOX relies on energy-dependent caveolae-mediated transcytosis. The *in vitro* tumor transcytosis of Rod JBNps–DOX was further investigated by delivering them to the ovarian cancer spheroids. As depicted in Fig. 5H, Rod JBNps were successfully delivered to cells inside the SKOV3 spheroids, in contrast to the peripheral delivery of Doxosomes and Non-rod JBNps.

Further functional assays of Rod JBNp delivery into SKOV3 cancer cells and spheroids were performed using Caspase 3/7 staining. Caspase 3/7 activity significantly increased after 24 h of treatment of SKOV3 cells with Rod JBNps, compared with that of an untreated control (Fig. S14). Then, as shown in Fig. S15A and Fig. S15B, following 24 h of treatment with Rod JBNp, annexin V-FITC/propidium iodide (PI) double staining analysis indicated that the proportion of early apoptotic SKOV3 cells increased to 73.6 %. We then demonstrated that the Rod JBNps achieved a significantly higher rate of apoptosis in a timedependent manner (Fig. S15C and Fig. S15D). To further test the functional delivery of DOX, we tested the formation of cancer spheroids upon the delivery of Rod JBNp. CLSM images and quantitative analysis of spheroid size revealed that Rod JBNp inhibits the initial formation of ovarian carcinoma spheroids better than in the controls (Fig. S17). Finally, we evaluated the apoptotic phenotype in SKOV3 tumor spheroids. As shown in Fig. 5I, treatment of the spheroids with Rod JBNp–DOX led to a significant increase in the number of apoptotic cells (Caspase 3/7 dye: red) and even indicated an apoptotic signal deep in the center of the spheroid compared to the Non-rod JBNp–DOX and Doxosome. High throughput flow cytometry analysis was performed to quantify the apoptotic signal in the spheroid (Fig. S18). The results revealed that the Rod JBNp–DOX achieved apoptotic efficiency of 61.8 %, which was higher than the rates for the DOX-free base, Doxosome, and Non-rod JBNp groups (9.8 %, 11.4 %, and 21.2 %, respectively) (Fig. 5J). This may be attributable to the rod-shaped morphology of the Rod JBNp, which allows the nanoparticles to penetrate further into the spheroid and deliver the DOX at a site deeper into its interior.

### 2.5. In vivo biodistribution and tumor penetration

**Fig. 6.**
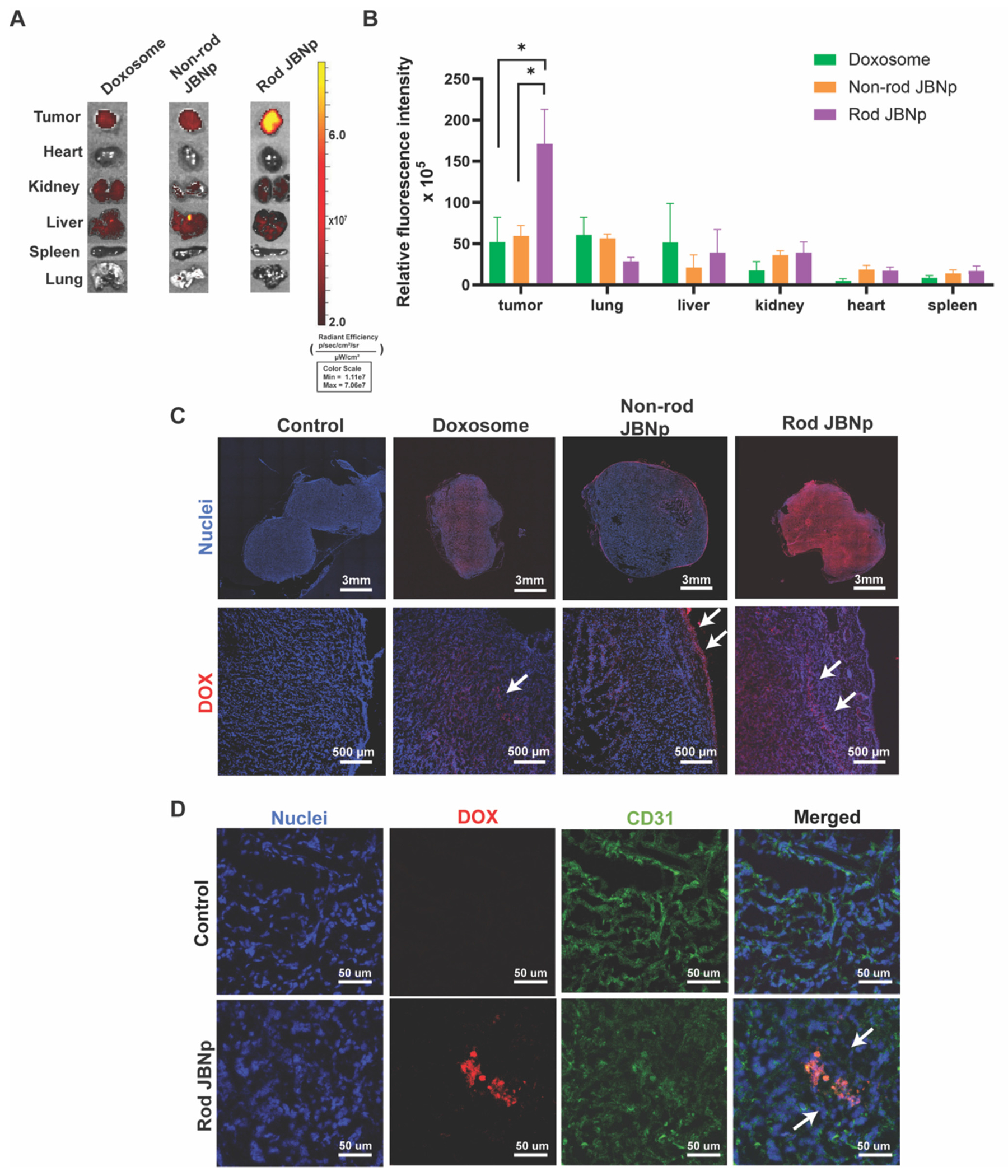
Rod-shaped JBNps showed tumor-specific biodistribution and improved infiltration through the tumor ECM. (A) *Ex vivo* biodistribution of major organs and tumors after intravenous injection to SKOV3 tumor xenograft NU/J mice for 72 h. (B) *Ex vivo* quantification of relative fluorescence intensity in major organs and tumors. (C) Tumor section and stained with DAPI, Blue indicates nuclei staining, Red indicates DOX. (D) Rod JBNp localization in tumor tissue near tumor blood vessels detected by the CD31 biomarker. Red: DOX; Blue: DAPI staining Green: CD31. The animal was sacrificed at 72 h post-injection. Error bars represent mean ± SEM (*n* = 5). (For interpretation of the references to colour in this figure legend, the reader is referred to the web version of this article.)

Given that rod-shaped JBNps exhibit superior transcytosis and penetration across densely packed tumor spheroids, we aimed to evaluate their potential for tumor penetration and their biodistribution *in vivo*, using an ECM-rich SKOV3 adenocarcinoma tumor xenograft mouse model. We investigated the *in vivo* biodistribution of Rod JBNps after intravenous injection to determine their tumor specificity and delivery efficiency. The biodistribution of Rod JBNps was evaluated in nude mice bearing a subcutaneous SKOV3 xenograft *via* intravenous infusion. *Ex vivo* fluorescence intensity images were obtained for the tumors and significant organs to explore the distribution of DOX. As depicted in Fig. 6A, Rod JBNps showed enriched distribution to the tumors compared to other major organs, as indicated by more intense fluorescence signal in the tumors. These results were further confirmed by quantitative analysis (Fig. 6B). Delivery by the Rod JBNps demonstrated significantly higher accumulation of DOX than the administration of Doxosome or Non-rod JBNps. Furthermore, to confirm the successful delivery of DOX, the tumor tissues were collected and sectioned for fluorescence examination 72 h after injection (Fig. 6C). Imaging of the sections revealed the greater efficiency of infiltration of Rod JBNps into the tumor compared with the administration of Doxosome or Non-rod JBNps. This is likely due to their rod shape, as Rod JBNp have improved the enhanced permeability and retention (EPR) effect and infiltration into the tumors [19,20]. Additionally, the distribution of DOX in tumors was analyzed *via* anti-CD31 immunohistochemistry. Confocal imaging of the stained sections revealed that DOX delivered by Rod JBNp carriers was mainly distributed to the CD31-stained blood vessels in the tumor region (Fig. 6D). By the passive targeting of tumors *via* the penetration effect due to their rod-shaped morphology, Rod JBNps have the potential to increase the efficacy of drugs while reducing the nonspecific distribution of drugs during the *in vivo* delivery process.

### 2.6. Antitumoral effect of rod-shaped JBNps to treat SKOV3 tumorbearing mice

**Fig. 7.**
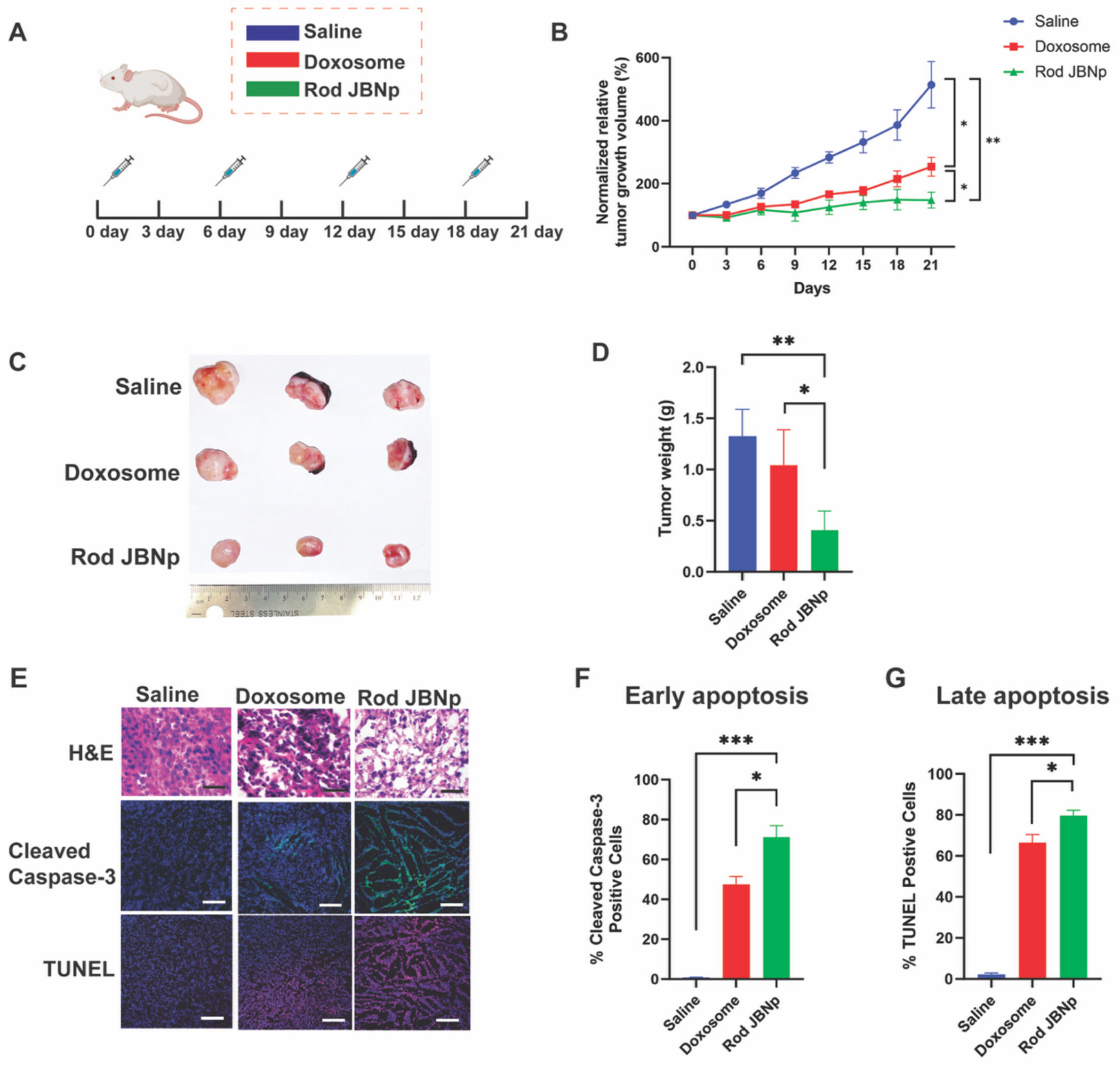
Rod-shaped JBNps induced significantly higher antitumor activity compared to spherical Doxosomes. (A) Schematic illustration of the treatment plan. (B) Relative tumor-growth curves during the treatment were measured by a caliber (*n* = 6). (C) Photograph of excised tumors from SKOV3 tumor-bearing mice after various treatments. (D) Weight of excised tumor. (E) Histopathological analysis of the tumors by hematoxylin and eosin (H&E) analysis (top), IHC staining of cleaved caspase-3 (middle), and TUNEL analysis (bottom) of the tumors, scale bar: 100 μm. (F) Quantitation of cleaved caspase-3 positive cells. (G) Quantitation of apoptotic cells from TUNEL staining. Percentages of cleaved caspase-3 positive cells and TUNEL-positive were quantitated by counting 100 cells from 5 random microscopic fields. Data are presented as the mean SEM, *n* = 5. ** *p* < 0.01, *** *p* < 0.001.

Based on the encouraging results of our biodistribution study, we further investigated the *in vivo* antitumor efficacy of Rod JBNps. When the tumor volume in the SKOV3 tumor-bearing nude mouse model reached approximately 200 mm^3^, these mice were randomly divided into three groups: saline, Doxosome, and Rod JBNps. They were then intravenously injected with one of these substances every 3 days for 21 days. Tumor volume was recorded every 3 days. After the treatments, all mice were euthanized, and collected tumors were weighed and used for quantitative analysis of the inhibition of tumor growth (Fig. 7A). As displayed in Fig. 7B, the tumors in the saline and Doxosome groups grew rapidly. In contrast, the growth of those in the Rod JBNp group was significantly inhibited during 21 days of treatment, indicating that Rod JBNps are effective for treating cancer. The tumors were then excised, photographed (Fig. 7C), and weighed. Compared with the findings in the saline group, Rod JBNp treated mice showed a 59.2 ±10*.*2% decrease in tumor weight (Fig. 7D). Moreover, the H&E staining results of tumor sections revealed increases in necrosis and apoptosis in tumor cells of the Rod JBNp group, compared with that observed in other groups. To further investigate the role of apoptosis in the effects of DOX, tumor sections were stained for the expression of cleaved caspase-3 and TUNEL to detect those cells at early and late stages of apoptosis, respectively (Fig. 7E). Consistent with the reduction in tumor growth, there was also a significant increase in the expression of the early apoptosis marker, cleaved caspase-3, compared with the finding upon treatment with Doxosome (Fig. 7F). Tumors treated with Rod JBNps also showed a significantly higher intensity of green fluorescent TUNEL staining, indicating significantly more late apoptotic cells than in tumors from the other groups (Fig. 7G). To rule out any effect of inflammation on apoptosis, we also confirmed that the proinflammatory responses in the tumor microenvironment do not play a role in the response of these tumors (Fig. S21). In conclusion, Rod JBNps were able to deeply penetrate the tumor, deliver DOX there, and induce significantly higher antitumor activity than Doxosome.

### 2.7. In vivo safety profile of rod JBNps

**Fig. 8.**
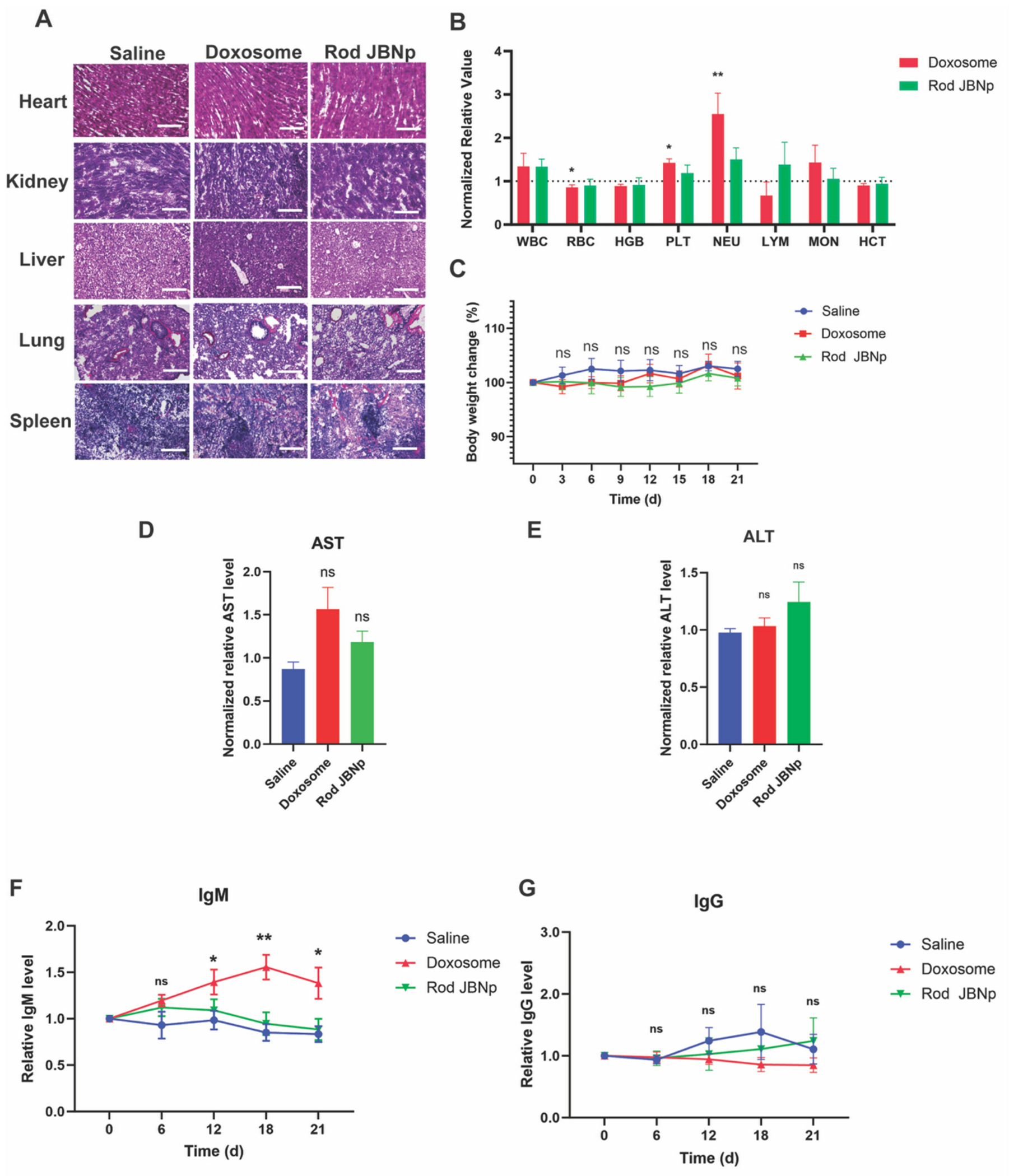
Rod-shaped JBNps had a better safety profile than spherical Doxosomes. (A) Major organs were obtained for H&E staining. Scale bar = 100 *μ*m. (B) Complete blood count (CBC) of Rod JBNP; Normalized relative value to before injection: white blood cells (WBC), red blood cells (RBC), hemoglobulin (HGB), platelet (PLT), neutrophil (NEU), lymphocytes (LYM), monocytes (MON), and hematocrit (HCT). (C) Body weight change of mice in the three treatment groups. (D) Serum levels of aspartate aminotransferase (AST). (E) Alanine aminotransferase (ALT) analysis. (F) Serums were obtained to detect inflammatory factors Immunoglobulin M (IgM). (G) Immunogobulin G (IgG). Data are shown as mean ± SEM (n = 5 per group), *, *p* < 0.05; **, p < 0.01; ***, p < 0.001. (For interpretation of the references to colour in this figure legend, the reader is referred to the web version of this article.)

Finally, we evaluated the safety of treatment with Rod JBNps. This included histopathological analysis of organs including the heart, kidney, liver, lung, and spleen. No histological differences were observed in these organs in mice from the Rod JBNp group compared with the findings in mice from the saline group, suggesting that Rod JBNps did not induce notable damage to these organs (Fig. 8A). Moreover, blood was collected for a complete blood count (CBC), which included white blood cell (WBC), red blood cell (RBC), hemoglobin (HGB), platelet (PLT), neutrophil (NEU), lymphocyte (LYM), monocyte (MON), and hematocrit (HCT) analyses. Comparisons were made relative to the preinjection baseline for mice in each group. Differential CBC analysis revealed that the Rod JBNps did not induce changes in the levels of any of the above-mentioned blood components, while Doxosome caused some significant changes, such as increases in NEU and PLT levels (Fig. 8B). The Rod JBNp group exhibited an average body weight during the treatment stage (Fig. 8C). In addition, serum blood biochemistry parameters in the form of serum aminotransferase (ALT) and aspartate aminotransferase (AST) were also analyzed to assess liver toxicity. After Rod JBNp treatment, there were no significant changes in ALT or AST levels compared with the findings in the saline group (Fig. 8D and Fig. 8E). We also assessed serum immunoglobulin M (IgM) and immunoglobulin G (IgG) antibody levels after treatment relative to the pre-injection baseline levels *via* enzyme-linked immunosorbent assay (ELISA). Although treatment with Doxosome resulted in elevation of the adaptive immune responsive cytokine IgM, the Rod JBNp treatment regimen did not increase IgM or IgG levels (Fig. 8F and Fig. 8G). Together, these results demonstrate that Rod JBNp treatment has a significantly better safety profile than treatment with Doxosome.

## 3. Discussion and conclusion

Among the various nanoparticle shapes explored for drug delivery, rod-shaped nanoparticles have demonstrated superior efficiency in penetrating tissues [5,21]. Existing rod-shaped systems, including gold nanorods or carbon nanotubes, suffer from poor biodegradability and typically require covalent functionalization for drug loading, limiting both their cargo versatility and biocompatibility [6–8,22]. In contrast, JBNps represent a new class of biomimetic, biodegradable nanorods capable of noncovalent drug encapsulation. They exhibit low cytotoxicity and immunogenicity while being high versatile in their ability to deliver a range of drug cargos, including small molecules, siRNA, and mRNA. Similar to LNPs, JBNps achieve high drug-loading efficiency, yet they offer the added advantage of a rod-like shape that facilitates deeper penetration into solid tissues, including tumors with dense ECM.

This shape advantage is particularly relevant in the context of tumors such as ovarian adenocarcinoma, where ECM density and receptor heterogeneity can hinder targeted delivery using antibody-drug conjugates (ADCs) [14,15,23,24]. In such cases, relying solely on molecular recognition can be limiting. The incorporation of physical design features like shape offers a receptor-independent strategy to enhance intratumoral accumulation. Our data herein support previous findings that rod-shaped nanoparticles exploit the enhanced permeability and retention (EPR) effect more effectively than spherical particles [25–27]. This approach can therefore bypass the challenges posed by inter-patient variability in surface marker expression, offering a broadly applicable passive targeting solution. Despite this study focuses on using phyiscal properties of nanoparticles for tumor targeting, we recognize that chemical properties can also strongly impact targeting ability of nanoparticle [28,29]. For example, we recently found that current type of JBNps with lysine side chains exhibit high binding affinity for integrin beta-1 (ITGB1) in the ECM, which may contribute to their targeting of ovarian tumors that overexpress ITGB1 (Fig. S20). As a future direction, surface chemistry of JBNps can be further studied and developed for tumoral targeting. Thus, integrating physical characteristics with chemical modifications in future designs may enhance targeting precision and therapeutic efficacy, paving the way for innovative approaches to treating challenging solid tumors.

Beyond geometry, the chemical composition and assembly mechanism of JBNps also contribute to their delivery potential. Unlike DNA origami or conventional polymeric nanocarriers, JBNps are assembled from small molecules *via* predictable noncovalent interactions. These include hydrogen bonding and π–π stacking, which enable the formation of a structurally stable yet biodegradable nanomaterial. The unique DNA-inspired chemistry confers enzymatic resistance while maintaining excellent biocompatibility, differentiating them from both rigid carbon nanotubes and enzymatically degradable nucleic acid-based carriers [30]. However, because the JBNp delivery system is based on dynamic self-assembly, it requires thorough investigation of colloidal stability, especially when compared to structurally rigid nanostructure such as gold nanoparticles and carbon nanotubes. As a potential solution, the side chains of JBNps can be modified to mediate their surface properties and enhance their stability.

Using a computationally guided design framework, we identified and validated key structural parameters by SAXS, MD simulations and RSM to optimize cargo loading. This predictive approach eliminated the need for extensive empirical screening and has the potential to offer a scalable strategy for designing next-generation carriers [31–34]. Importantly, lysine-functionalized JBNps showed favorable energetics for cargo intercalation under physiological conditions, supporting efficient delivery.

Crucially, the rod-shaped JBNps fabricated here (AR ~5.3) outperformed spherical liposomal Doxosome (AR ~1) in both tumor penetration and therapeutic efficacy. Not only did Rod JBNps deliver DOX more effectively, but they also exhibited favorable biocompatibility profiles. While Doxosome administration elevated neutrophil and platelet counts, which are side effects often associated with anthracyclines [35,36], Rod JBNps preserved normal hematologic parameters. This distinction is clinically meaningful given that increased neutrophil counts post-DOX treatment have been linked to cardiotoxicity and vascular damage [37]. Furthermore, repeated dosing of Doxosome elicited IgM production, potentially enhancing opsonization and accelerating clearance [38,39], whereas Rod JBNps showed minimal immunogenicity, likely due to their biodegradable and noncovalent design.

While the enhanced performance of Rod JBNps was demonstrated in a proof-of-concept ECM-rich adenocarcinoma model, further *in vivo* studies across different tumor types and dosing strategies are needed to validate their generalizability before they can be considered a one-size-fits-all solution. Additionally, further investigations are required to delineate the relative contributions of physicochemical properties and underlying mechanisms that influence circulation time, biodistribution, or immune evasion.

In summary, this study highlights the crucial role of nanoparticle shape in enhancing targeting for hard-to-penetrate solid tumors through the development of a new family of rod-shaped delivery vehicles—Rod JBNps. It also demonstrates a computation-aided methodology to refine drug loading and design parameters, providing a broadly applicable framework for future nanoparticle engineering. These findings offer a blueprint for engineering the next generation of nanoparticles capable of delivering therapeutics to otherwise inaccessible tissues, with potential for broad clinical application in oncology and beyond.

## 4. Experimental section/methods

### 4.1. Materials and methods

JBNt monomer was synthesized according to previously reported procedures and purified using high-performance liquid chromatography (HPLC) [40]. Doxorubicin hydrochloride (DOX⋅HCl) was purchased from TCI America. DOX free base was purchased from MedKoo Bio-sciences. Tert-butanol (*t*-BuOH) was purchased from Alfa Aesar. Methanol-*d*_4_ (CD_3_OD) was purchased from ACROS Organics. NaOD in D_2_O was purchased from Sigma Aldrich. Fibroblast, SKOV3, and MCF-7 cells were obtained from ATCC. DMEM cell culture medium (Gibco), Trypsin-EDTA (Gibco, 0.25 %), Fetal Bovine Serum (FBS, Gibco), Non-essential Amino Acids Solution (100×, Gibco), Phosphate Buffered Saline (PBS, Gibco), Ethanol (70 % solution) and, Poly-l-lysine (PLL, P8920, Sigma-Aldrich), Polyethylenimine (PEI, CAS#: 9002-98-6, Alfa Aesar), Single-Walled Carbon Nanotube (SWCNT, US Research Nanomaterials, US4100W), Lipofectamine 2000 were obtained from Thermo Fisher Scientific. Triton X-100 (Invitrogen^™^, 1.0 %), DAPI, and 4 % formaldehyde (Invitrogen) were purchased from Fisher Scientific. Single-walled carbon nanotubes dispersion (SWCNs) was purchased from US Research Nanomaterials. Polyethylenimine (PEI, branched, MW 70000, 30 % *w*/*v* in water) was purchased from Alfa Aesar, Cell Counting Kit-8 (CCK8) was purchased from Dojindo, and Poly-l-lysine (PLL, 0.1 % w/v in water) was purchased from Sigma. 24-well plate (Corning) and 96-well plate (Corning) were purchased from Fisher Scientific, of which catalog numbers are 07–200-740 and 07–200-91, respectively.pectively.

### 4.2. Computation-aided model setup

MD simulations were conducted for two computation model systems. The first model aimed to establish the optimal condition regarding the relative positioning of monomers within the ring. This model was implemented in a simulation box of 10.0 × 10.0 × 7.2 nm^3^ consisting of water molecules having a density of 1.0 g cm^−3^. Subsequently, two G^C Janus base monomers were randomly inserted into the box under neutral conditions with their ring plane parallel to *x-y* plane, and the out-of-plane torsion motion was restrained. The outcome of this model was to describe the 2D-potential energy surfaces of relative stacking energies between two G^C Janus base monomers. The second model aimed to determine the energy of DOX loading to the different JBNt structures. The system included a simulation box of 10.0 × 10.0 × 7.2 nm^3^ composed of water molecules having a density of 1.0 g cm^−3^, a single DOX molecule, and a JBNt structure with/without chloride ions. Four different JBNt structures were used in this model, including Gly-JBNt, Asp-JBNt, and neutral/acidic Lys-JBNt. The JBNts were initially made up of 16 layers along the *z*-direction with a layer spacing of 4.5 Å, in which six G^C Janus-based monomers assembled each layer to form a ring (Fig. S1A, i–ii, Fig. S1B, i–ii, Fig. S2A, i–ii, Fig. S2B, i–ii). To study the effect of pH values on the delivery of DOX to the JBNt, a neutral form of lysine-based monomer (Fig. S2A) and a neutral DOX molecule (Fig. S3A) was implemented in the high pH condition. Under acidic condition (low pH), the protonated lysine-based ring (Fig. S2B) and protonated DOX form were introduced (Fig. S3B). Additionally, chloride ions were used to neutralize the system with protonated structures before running the DOX loading simulations.

### 4.3. MD simulation setup

All-atom MD and metadynamics simulations were performed using GROMACS package with CHARMM27 force field, and TIP3P explicit solvent model at room temperature (300*K*) [41–44]. For each system, the steepest descent algorithm was first employed to minimize the whole system within 50,000 steps. Before the production runs, the sequential equilibrium processes in 0.5 ns-NVT (constant number of atoms, volume, and temperature), 0.5 ns-NPT (constant number of atoms, pressure, and temperature), and 1.0 ns-NVT ensembles were performed to adjust the systems into the desired temperatures, volumes, and pressure. To fully relax the JBNts before implementing DOX loading process, an additional 10.0 ns production run was performed under NPT ensemble. The leap-frog integrator produced an unbiased MD trajectory for each system with an integration time-step of 1 fs under different ensemble conditions. Parrinello-Rahman barostat was employed to control the pressure at 1 bar with a coupling constant of 2 ps [45]. V-rescale was used to monitor the system temperature at 300 K with a time constant of 1 ps, a modified Berendsen thermostat [46]. The particle Mesh Ewald method was employed to consider the electrostatic interactions with a real-space grid distance of 1 nm [47]. The cutoff of non-bonded interactions, including electrostatic and van der Waals forces, was set to 1.0 nm. The SETTLE algorithm was used to constrain water molecules and all non-water bonds were constrained using the LINCS algorithm [48]. For metadynamics, the out-plane torsion angle and vertical distance between two G^C Janus based monomers were restrained. All simulations were conducted under periodic boundary conditions. Snapshots during the simulation were rendered using the Visual Molecular Dynamics (VMD) software [49]. To model the system under a low-pH environment, we adopted protonated lysine-based rings (Fig. S2B, i–ii) and protonated forms of DOX (Fig. S3B, i–ii). Chloride ions were introduced to neutralize the system upon introducing protonated Lys-JBNt and protonated DOX into the box, thus generating the required low-pH mimicking condition for the study. Following this, PMF calculations were implemented to examine the loading of DOX onto the protonated Lys-JBNt. As previous PMF calculations for Lys-JBNt were conducted under neutral conditions (pH = 7), our current focus is on evaluating the capacity of DOX loading onto the Lys-JBNt under acidic conditions.

### 4.4. Metadynamics simulations

Metadynamics is an improved sampling technique used to explore the free energy profile of a system along the reaction coordinates, usually named collective variables (CVs), which can speed up the system out of a local free energy minimum and traces the other free energy minima by intermittently adding a history dependent biasing potential energy that impels the dynamics to explore those undiscovered conformations [50–53]. Compared with the motion equation of MD simulations, metadynamics has the additional forces imposing on atoms originating from the history dependent biasing potential, which can be accumulated as Gaussian functions,

(1) $$V_{B}(s(x),t)=w\sum_{t\prime =\tau_{G},2\tau_{G},3\tau_{G}\cdots }exp\left(-\sum_{i=1}^{M}\frac{{\left(s_{i}(x)-s_{i}\left(t\prime \right)\right)}^{2}}{2\delta_{i}^{2}}\right)$$

where *s_i_* (*x*) is the *i*^th^ of *M* reaction coordinates or collective variables. The constant *w* and *δ_i_* are respectively the height and width of the Gaussian function adding at a constant time intervals of $\tau_{G}$. Well-tempered metadynamics is another improved metadynamics, which corresponds to changing the constant height of the Gaussian function as a time-dependent variable in eq. 1, expressed as,

(2) $$V_{B}(s(x),t)=w_{0}e^{-\frac{V_{b}\left(s,t\prime \right)}{\Delta Tk_{b}}}\sum_{t\prime =\tau_{G},2\tau_{G},3\tau_{G}\cdots }exp\left(-\sum_{i=1}^{M}\frac{{\left(s_{i}(x)-s_{i}\left(t\prime \right)\right)}^{2}}{2\delta_{i}^{2}}\right)$$

where *w*_0_ defines the initial height of Gaussian functions and Δ*Tk_b_* is a characteristic energy [54]. During the simulation evolution, more and more Gaussians functions are summed up and not updated until the full energy landscape is explored. Thus, over a long simulation time, the exact free energy can be evaluated from the converged biasing potential as

(3) $$F^{w}(S)=-\frac{\Delta T+T}{\Delta T}\underset{t\to \infty }{lim}V_{B}(s(x),t)=\frac{B}{b-1}\underset{t\to \infty }{lim}V_{B}(s(x),t)$$

where B=(T+ΔT)/T is a scaling factor from a higher temperature T+ΔT to the normal temperature *T*.

In this work, to obtain the 2D free energy profiles, we performed the metadyamics simulations [50–52] for the first system using PLUMED 2.7 which is patched to GROMACS 2021 [55,56]. Two collective variables were chosen to investigate JBNt self-assembly. A collective variable (CV1) was defined as the vertical distance between two G^C Janus base monomers, and another collective variable (CV2) was considered the relative torsion angle between two G^C Janus base monomers. We analyzed the variation in stacking energies as a function of the vertical distance and the relative torsion angle between two G^C Janus base monomers within a neutral condition. The Gaussian widths were set to 0.05 for CV1 and 0.05 for CV2 with the initial height of W = 0.5 kJ/mol added by every 20 ps. The grid bounds were set to 0–7.0 nm for CV1 and −π ~ +π rad for CV2. The grid bins were divided into 500 for distance space and 600 for dihedral space. To avoid overfilling, the biasfactor parameter was set to 10 at temperature of 300 K, and the Gaussian deposit time *τ_G_* was set to 1 ps. A total simulation time of 20 ns was performed for metadynamics to ensure the convergence of the free energy profile.

### 4.5. The potential of mean force

PMF calculations were performed for the second system according to the literature using GROMACS [57]. During the delivery process of DOX, the PMF was calculated as a function of DOX-JBNt distance using the umbrella sampling method [58]. The JBNt was placed in a simulation box of 10.0 × 10.0 × 7.2 nm^3^. Water molecules with a density of 1.0 g cm^−3^ were filled into the box. Subsequently, we ran a 10 ns NPT equilibrium to fully relax the JBNt (Fig. S1A, iii, Fig. S1B, iii, Fig. S2A, iii, Fig. S2B, iii). After that, a single DOX molecule was introduced into the simulation box, followed by the PMF simulation of DOX loading to each of JBNt structures. A harmonic potential was acted on the center of mass of DOX molecules at the *i*^th^ window along the radial direction of JBNts, and a series of separate umbrella simulations were performed. The harmonic potentials are represented by $u_{i}\left(x_{i}\right)=\frac{1}{2}k\left(x-x_{i}\right)$, where *k* is the force constant, *x* is the coordinate of DOX along the radial direction, and *x_i_* is the center position of the *i*^th^ window. In this work, the force constant was set to 1,000 kJ/mol nm^−2^, and the elongation rate of −0.3 nm ns^−1^ was chosen as the imaginary spring attached to the pull groups. The initial center position of DOX was located about 4.0 nm from the axis center of JBNt, and the final position was located about 0.5 nm from the axis center of JBNt. Window space was chosen at 0.05 nm to ensure the overlapping of the histogram of the positions of JBNt throughout the pulling direction (Fig. S4) such that a continuous free energy profile could later be derived from these simulations. For each window, an NPT equilibrium run was conducted for 5 ns, followed by a production run of 5 ns for the PMF calculations. The weighted histogram analysis method (WHAM) was employed to carry out the result analysis [59]. Statistical errors were estimated with bootstrap analysis using 200 bootstraps and 50 bins for the PMF profiles [60].

### 4.6. Small Angle X-ray Scattering (SAXS)

Two-dimensional SAXS measurements were conducted at Brookhaven National Laboratory, utilizing the 16ID-LiX Beamline, which possesses a synchrotron energy of 13.5 keV. Two aqueous solutions of JBNt (10 mg/mL) were prepared at neutral pH in the absence and presence of DOX. The samples were loaded in glass cells and maintained at 20 °C during the measurements over an exposure time of 3 s. Radial integration was carried out to yield 1-D reduced data, the scattered intensity as a function of the scattering vector, $q\left(\frac{4\pi }{\lambda }sin\frac{\theta }{2},\theta \right.$ and *λ* are the scattering angle and wavelength, respectively) between 0.006 and 3.2 Å^−1^. The reduced 1-D data was corrected by the background subtraction of water scattering in the same cell. The corrected data were further best fitted using a combined model of core-shell cylindrical (CSC) morphology and a Gaussian peak, within the *q* range of 0.006 to 1.3 Å^−1^.

### 4.7. Response Surface Methodology (RSM)

The following factors were analyzed at these discrete values: pH: 2.9, 6.5, 8.3; Temperature (°C): 4 °C, 25 °C, 38 °C; Time (hours): 0.5, 5, 24, 48, 96. From the experimental results, a second-order polynomial function was generated to model the relationship between independent variables pH (X1), Temperature (X2), and Time (X3) to the dependent variable, Encapsulation Efficiency (Y):

$$Y=\beta_{0}+\beta_{1}X_{1}+\beta_{2}X_{2}+\beta_{3}X_{3}+\beta_{11}X_{1}^{2}+\beta_{22}X_{2}^{2}+\beta_{33}X_{3}^{2}+\beta_{12}X_{1}X_{2}+\beta_{12}X_{1}X_{3}+\beta_{13}X_{1}X_{3}+\beta_{23}X_{2}X_{3}$$

Where *β*_0_ represents the intercept, *β*_1,2,3_represent the principal effects of each factor, *β*_11,22,33_represent the squared effects of each factor, and *β*_12,13,23_represent the interaction effect between factors. Statistical analysis, including ANOVA and multiple response prediction, was performed to optimize the model’s response (Table S1 and Table S2). The R2 of the model was 89.49 %, with an adjusted R2 of 86.78 %, and a predicted R2 of 82.30 %. Further, the S (standard deviation of the residuals) of the model was 0.0932. This is substantially lower than the standard deviation of the observed response values, 0.256. Thus, it can be concluded this model has a strong fit to the experimental data (Table S3). A 4D scatterplot was created to visualize the experimental data. The creation of this regression model, its corresponding statistical analysis, and the 4D experimental scatterplot were produced with Minitab ^®^ Version 21.1 (Minitab LLC, State College, PA). An additional 4D scatterplot was created, using the RSM model instead of experimental values, in Wolfram Mathematica version 13.2 (Wolfram Research, Champaign, IL). Three 3D surface plots were generated from the RSM, each demonstrating the relationships between two factors and the response. These were created in OriginPro 2024, Package 10.1.0.170 (OriginLab, Northampton, MA).

### 4.8. Fabrication of JBNt-DOX

200 μg DOX free base was added into 1 mL JBNts solution (1 mg/mL) and vibrated with a LP Vortex Mixer (Thermo Scientific) for 24 h. NaOH was used to adjust the pH. The solution was aged for another 1 day to allow for the drug-loading process. Then, the JBNt-DOX suspension was divided evenly into two parts. One part of the suspension was named as the JBNt-DOX group for the test directly. Another part of the suspension was sonicated with a sonicator (Qsonica) for 2 mins 30 s (20 kHz, 500 W). Then, it was named the Rod JBNp group. Another 100 μg DOX-free base was added into 0.5 mL PBS and vibrated with an LP Vortex Mixer (Thermo Scientific) for 24 h as well. The suspension was then centrifuged in a Corning Mini Centrifuge for 20 s.

### 4.9. Nuclear magnetic resonance experiments

JBNt monomer was first dissolved in CD3OD and aged for 1 day. t-BuOH was added as an internal standard. DOX·HCl was added in a 1:10 (DOX: JBNt monomer) mole ratio and NaOD in D_2_O was used to adjust the pH. Samples were measured using the Bruker AVANCE 500 spectrometer at different temperatures and timepoints. Drug loading rates were calculated by peak integrations of DOX monitored at 5.5 ppm while calibrating the internal standard’s peak integration to 1 (Table S4).

### 4.10. Characterization of rod JBNp

*S*amples were measured using the Malvern Zetasizer Nano ZS90 and Nanodrop OneC microvolume UV–Vis spectrophotometer for DLS and UV–Vis experiments. To assess the stability of Rod JBNps, UV–Vis absorbance spectra were recorded over a 14-day period. Nanoparticle samples were stored at room temperature in PBS (pH 7.4), and measurements were taken at predetermined time points (0–14 days). For TEM, 3 μL of the sample was loaded on a 300-mesh copper grid, negatively stained with 50 μL 0.5 % uranyl acetate, and dried using filter paper. TEM images of JBNt-DOX fabricated with varying sonication power and time were captured by the FEI Tecnai 12 G2 Spirit BioTWIN microscope at 120 kV (Fig. S9). The agarose gel electrophoresis assay was conducted using a 0.8 % low-melting agarose gel followed by electrophoresis at 100 V for 60 min.

### 4.11. Apparent pK_a_ assay

The apparent *pK_a_* of Rod JBNp was determined using a fluorescent probe 2-(p-toluidino) naphthalene-6-sulfonic acid (TNS). In brief, the TNS reagent (Sigma) was prepared as a 100*μM* stock in RNase free water. Rod JBNp were diluted in 90 *μ*L of buffered solutions (triplicates) containing 10 mM HEPES, 10 mM 4-morpholineethanesulfonic acid, 10 mM ammonium acetate, 130 mM NaCl, where the pH ranged from 2.71 to 11.5. 10 *μ*L of stock TNS was added to the Rod JBNp solutions and mixed well in a black 96-well plate. Fluorescence intensity was monitored in a microplate reader (Molecular Devices) using excitation and emission wavelengths of 321 and 447 nm. A sigmoidal plot of fluorescence *versus* buffer pH was created with the resulting fluorescence values A sigmoidal best-fit analysis was applied to the fluorescence data of each Rod JBNp. The pKa was calculated as the pH value at which half of the maximum fluorescence is observed.

Cells and Spheroids Culture: Human fibroblast (ATCC) and SKOV3 cells were cultured on T75 flasks (Fisher Scientific) at 5 % CO2 at 37 °C using McCoys 5 A media (Thermo Fisher Scientific) supplemented with 1 % l-glutamine and 10 % FBS. To make the SKOV3 spheroids, SKOV3 cells were seeded on 96-well Nunclon Sphera round bottom plates (Thermo Fisher Scientific) at a density of 2 ×10^3^ cells/mL. Cells were incubated for 5 days to achieve compact and consistent spheroids before subsequent experiments.

### 4.12. In vitro cytotoxicity assay

JBNts were dissolved in nuclease-free water, SWCNTs were supplied as an aqueous dispersion and further diluted in water, and branched polyethylenimine (PEI; 30 % *w/v* aqueous solution) was diluted in Opti-MEM. Poly-l-lysine (PLL; 0.1 % w/v in H_2_O) was prepared in nuclease-free water. Each of these stock solutions (JBNts, SWCNTs, PEI, and PLL) was then further diluted with cell culture medium to prepare final working concentrations of 10, 5, 1, and 0.2 μg/mL for transfection. Lipofectamine 2000 was prepared according to the manufacturer’s protocol, using Opti-MEM as the initial solvent, and subsequently diluted with cell culture medium to match the same final concentrations. 96–well plates were prepared for the cytotoxicity assay by seeding each well with 100 μL of fibroblast cell suspension (5,000 cells). Plates were incubated at 37 °C for 24 h to allow cell attachment, then treated with the test materials and co-incubated for an additional 24 h. Cell viability was assessed using the Cell Counting Kit-8 (CCK-8) assay, and absorbance at 450 nm was measured after 1 h using a SpectraMax M3 plate reader. Results were normalized to the negative control group, which did not receive any treatment.

### 4.13. In vitro drug delivery assay

Assembled Rod JBNp were immediately transferred to the SKOV3 cells and then incubated at 37 °C and 5 % CO_2_. The supernatant was used for the drug delivery test, which was named the Dox free base group. All three groups (Dox-free base, Non-rod JBNp, Rod JBNp) of materials were diluted 10 times before use. 250 μL cell suspension (1 × 10^4^ cells) was seeded into one well of the 8-well chambered coverglass. 5 wells of cells were prepared for the test. The cells were incubated in an incubator (37 °C, 5 % CO_2_) for 24 h. Each well of cells received 250 μL of materials. For the control group, the same volume of PBS was used instead of the materials. Then, the cells were co-incubated with materials for another 24 h. For siRNA delivery, 5 μM of AF488-tagged siRNA was co-assembled with Rod JBNp (with pre-loaded with DOX) and delivered to SKOV3 cells for 24 h. For mRNA delivery, 0.2 μg of Cy5-labeled eGFP-encoded mRNA was assembled with Rod JBNp and delivered for 72 h. After incubation, DAPI was used to stain the nucleus of the cells. Nikon A1R Spectral Confocal was utilized to obtain the confocal images and Image J was used to analyze the images. Spheroids were generated from SKOV3 and cultured for approximately 5 days before they reached a diameter of approximately 150 *μ*m. Assembled Rod JBNp were immediately transferred to SKOV3 spheroids and then incubated for 24 h at 37 °C and 5 % CO_2_. Then, spheroids were fixed with 4 % formaldehyde and stained with DAPI (overnight). A Nikon A1 confocal laser-scanning microscope was used for fluorescence imaging and was visualized using CLSM *Z*-stacks with 25um Z-intervals.

### 4.14. Cell uptake mechanism study

For cell uptake mechanism studies, SKOV3 cells were incubated with one of the following a single endocytic inhibitors under the specified conditions prior to nanoparticle exposure: chlorpromazine hydrochloride (Cpz, 100 μM for 30 min), methyl-β-cyclodextrin (Mβcd, 1 mM for 30 min), filipin (Fpn, 5 μg/mL for 1 h), genistein (Gen, 50 μg/mL for 1 h), sodium azide (NaN_3_, 100 mM for 30 min), or latrunculin A (Lat, 2 μM for 30 min). Following inhibitor treatment, cells were washed three times with PBS to remove residual inhibitors and prevent interference with subsequent nanoparticle uptake. The cells were then immediately exposed to Rod JBNp–DOX (final DOX concentration: 8 μg/mL) in complete culture medium and incubated for 24 h at 37 °C. Cellular uptake was quantified by flow cytometry (BD LSRFortessa X-20 Cell Analyzer, BD Biosciences) based on DOX fluorescence. To ensure that inhibitor treatments did not affect cell viability, a CCK-8 assay was performed on SKOV-3 cells treated under the same conditions, which confirmed no significant cytotoxicity (Fig. S13).

### 4.15. Apoptosis assay

Cells (5 × 10^5^ cells) grown on a 24-well plate for 24 h were treated with DOX-free base, Non-rod JBNp, or Rod JBNp. Upon washing twice with PBS, cells were digested with 1 % EDTA at 37 °C for 5 min. For the cancer spheroids, the harvested spheroids following treatment were further dissociated. The cells were collected by centrifugation at 1000 rpm for 4 min. Apoptotic cells were determined with an FITC Annexin V Apoptosis Detection Kit (BD Biosciences, USA) according to the manufacturer’s protocol. Briefly, Annexin V-FITC and propidium iodide (PI) were added to the SKOV3 cell suspension for 20 min at room temperature in the absence of light. Then, SKOV3 cells were analyzed on a Beckman Coulter CytoFLEX flow cytometry (Fig. S16). For Caspase 3/7 staining, apoptotic cells were determined with an Incucyte Caspase 3/7 Dyes kit. Then, activation of apoptosis using Caspase 3/7 dye (red) and nuclei (blue) was imaged by confocal laser microscope after treatment for 48 h.

### 4.16. Animal studies

The mouse studies were approved by the Institutional Animal Care and Use Committee of the University of Connecticut, Health Center, where the studies were performed under Protocol A-AP-200366-9224. Female 10- to 12-weeks-old NU/J (strain: 002019) mice from the Jackson Laboratory (Bar Harbor, ME, USA) were used for all mouse studies, including treatment, biodistribution experiments, with random assignment of mice to experimental groups. Mice were kept in a pathogen-free environment and fed at the conditions of 25 ± 2 °C and 55 % humidity with a 12 h light/dark cyclebyh guidelines established by the Institutional Animal Care and Use Committee (IACUC).

### 4.17. In vivo biodistribution study

Female 6- to 8- week-old NU/J mice were inoculated subcutaneously with 5 × 10^6^ SKOV3 cells supplemented with Matrigel (Corning, #354234) in a 1:1 volume ratio of cell suspension to Matrigel, and allowed to grow for 4 weeks until the tumor size was 200 mm^3^. Fluorescence imaging was performed using IVIS Spectrum Imaging system (PerkinElmer, USA) after mice were injected with 0.86 *mg*/kg of Doxosome, Non-rod JBNp (Dox-loaded), and Rod JBNp (Dox-loaded) (Fig. S19).

### 4.18. In vivo antitumor efficacy of rod JBNp in SKOV3 tumor models

After subcutaneous injection of 5 × 10^6^ SKOV3 tumor cells, tumors were allowed to grow for approximately 1 month until they reached a target volume of ~200 mm^3^. This tumor size was selected as the starting point for treatment to ensure a consistent baseline tumor burden across animals. Once tumors reached ~200 mm^3^, mice were randomly assigned to the following treatment groups (*n* = 6 per group): saline, Doxosome, and Rod JBNp. Group assignments were made to ensure similar tumor sizes across groups prior to the start of treatment, based on initial tumor measurements obtained using calipers. The experimenter measuring tumor size over time was blinded to group assignments. For SKOV3, mice (n = 6) were intravenously injected four times at three-day intervals with 150uL of treatment groups per mice per dose. All the treatments were administered *via* intravenous injection every three days for 21 days. Mice were treated with Doxorubicin dose of 0.86 *m*g/kg. Tumor growth curves were plotted based on the calculation of volume = ½ (length × width^2^).

### 4.19. Histological and immunofluorescence analysis

Tissue was collected and cryosections (8*μ*m) were prepared for immunofluorescence as described in references [61, 62]. The rabbit anti-CD31 and anti-active caspase antibodies were used to identify endothelial and caspase-3 activity, respectively. Incubation with primary antibody was done at 4 °C overnight with either anti-CD31 diluted 1:100, or anti-active caspase diluted 1:100. After primary antibodies, tissues were incubated with the goat anti-rabbit IgG labeled with AF488. A Click-iT Plus TUNEL Assay with Alexa 647 (Molecular Probes C10619) was used to examine the TUNEL staining. Fluorescence images were captured by Nikon A1R confocal microscope. Analysis was performed using Image J software. Cryosectioned tissues were stained with H&E for general histologic examination. And a board-certified veterinary pathologist performed blinded histopathological analysis on all sections.

### 4.20. Whole blood and serum chemistry analysis

Whole blood and serum were collected from the mice *via* the previously indicated timeline. The peripheral blood was collected in Mini-collect K2E K2EDTA blood sample tubes. IDEXX BioAnalytic analyzed collected samples for clinical chemistry and hematology of the peripheral blood. Results of the CBC studies are shown in Table S5. Serum levels of IgG, IgM, AST, and ALT were quantified using commercially available ELISA kits (Life Diagnostics, USA) following the manufacturer’s protocols. Briefly, 96-well plates pre-coated with capture antibodies were incubated with diluted serum samples at room temperature. After washing, wells were incubated with HRP-conjugated detection antibodies, followed by the addition of substrate solution. Absorbance was measured at 450 nm using a SpectraMax M3 microplate reader. Concentrations were calculated based on standard curves generated for each target analyte.

### 4.21. Statistical analysis

*S*tatistical analyses were performed using GraphPad Prism 7 software. Error bars were expressed as the mean ± SEM. Numerical data were analyzed *via* Student’s *t*-test, followed by ANOVA. *P* values <0.05 were considered significant.

## Supplementary Material

Supplementary information

## Data Availability

The data that support the findings of this study are available from the corresponding author upon reasonable request.

## Appendix A. Supplementary data

Supplementary data to this article can be found online at https://doi.org/10.1016/j.jconrel.2025.114169.
