# Supplementary material for: Computation-aided design of rod-shaped nanoparticles for tumoral targeting
Source: J Control Release. Author manuscript; Available in PMC 2026 Jul 20. (PMC13382647; doi:10.1016/j.jconrel.2025.114169)
Supplement: Supplementary information [file NIHMS2190955-supplement-Supplementary_information.pdf]

Computation-aided design of rod-shaped nanoparticles for tumoral targeting

†Jinhyung Lee[1], †Wuxia Zhang[1], Danh Nguyen[2], Libo Zhou[1], Justin Amengual[5], Jin Zhai[1], Trystin Cote[1], Maxwell Landolina[1], Elham Ahmadi[3], Ian Sands[1], Neha Mishra[4], Hongchuan Yu[7], Mu-Ping Nieh[5,6], *Kepeng Wang[3], *Ying Li[2], *Yupeng Chen[1]

[1]Department of Biomedical Engineering, University of Connecticut, Storrs, CT 06269, United States of America.

[2]Department of Mechanical Engineering, University of Wisconsin-Madison, Madison, WI 53706, United States of America.

[3]Department of Immunology, University of Connecticut Health Center, Farmington, CT 06030, United States of America.

[4]Department of Pathobiology and Veterinary Science, University of Connecticut Health Center, Farmington, CT 06030, United States of America.

[5]Polymer Program, Institute of Materials Science, University of Connecticut, Storrs, CT 06269, United States of America.

[6] Department of Chemical and Biomolecular Engineering, University of Connecticut, Storrs, CT 06269, United States of America.

[7] The Warren Alpert Medical School of Brown University and Rhode Island Hospital, Providence, RI 02903, United States of America.

† These authors contributed equally to the manuscript.

*Corresponding authors:

Dr. Kepeng Wang

Dr. Ying Li

Dr. Yupeng Chen

Keywords: Computation-aided design, Janus Base Nanomaterials, Drug Delivery, Nanorods, molecular dynamics

Supplementary Information Guide

Supplementary Figure 1. Simulation of self-assembly of Gly-JBNt and Asp-JBNt.
Supplementary Figure 2. Simulation of self-assembly of neutral and protonated Lys-JBNt.
Supplementary Figure 3. MD simulation of DOX.
Supplementary Figure 4. Histogram of the positions of JBNt.
Supplementary Figure 5. Experimental validations of JBNt-DOX.
Supplementary Figure 6. Deconvolution of JBNt and JBNt-DOX
Supplementary Figure 7. Small molecule (Resveratrol) loaded to JBNt.
Supplementary Figure 8. Stability assay of Rod JBNp.
Supplementary Figure 9. TEM images of Rod JBNp candidates.
Supplementary Figure 10. Fabrication and characterization of JBNt-DOX and Rod-JBNp.
Supplementary Figure 11. Time-dependent cellular delivery of Rod JBNp.
Supplementary Figure 12. Co-delivery of siRNA with DOX via JBNps.
Supplementary Figure 13. CCK-8 assay for inhibitor-treated cells
Supplementary Figure 14. Apoptosis assay of Rod JBNP after staining with Caspase 3/7.
Supplementary Figure 15. Time-dependent apoptosis assay of Rod JBNp.
Supplementary Figure 16. Flow cytometric gating of apoptosis assay.
Supplementary Figure 17. Formation of cancer spheroid upon Rod JBNps.
Supplementary Figure 18. Apoptosis assay of SKOV3 spheroids after treatments.
Supplementary Figure 19. Ex vivo biodistribution of nanoparticles in mice bearing SKOV-3 tumors.
Supplementary Figure 20. Analysis of Integrin beta-1 (ITGB1) binding to the JBNt.
Supplementary Figure 21. Quantification of proinflammatory cytokines in tumor.

Supplementary Table 1. Response surface methodology (RSM) model evaluation.
Supplementary Table 2. Coded regression coefficients for the RSM model.
Supplementary Table 3. Model summary statistics for RSM
Supplementary Table 4. Doxorubicin (DOX) loading rates at different pHs.
Supplementary Table 5. Result of the complete blood count (CBC) study in Rod JBNp.

Supplementary Figures

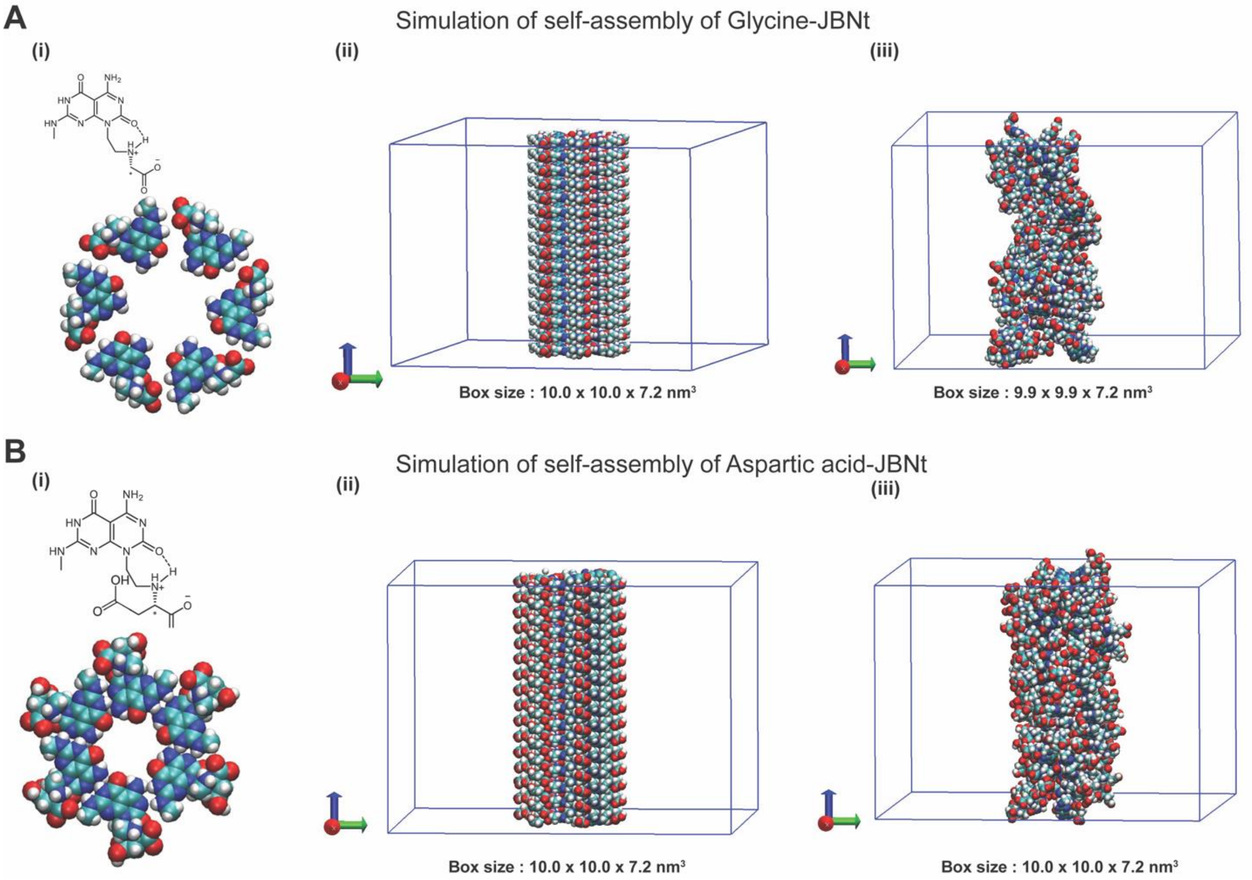

Figure S1. Simulation of self-assembly of Gly-JBNt and Asp-JBNt. (A, i) A ring built from six glycine-based monomers. (A, ii) 16 layers of glycine-based ring to form a JBNt with layer spacing of 4.5 Å. (A, iii) JBNt after 10 ns equilibration MD simulation, before loading DOX simulation. (B, i) A ring built from six Asp-based monomers. (B, ii) 16 layers of Asp-based ring to form a JBNt with layer spacing of 4.5 Å. (B, iii) JBNt after 10 ns equilibration MD simulation, before loading DOX simulation. Water molecules are included in the simulation, but not shown here for clarity.

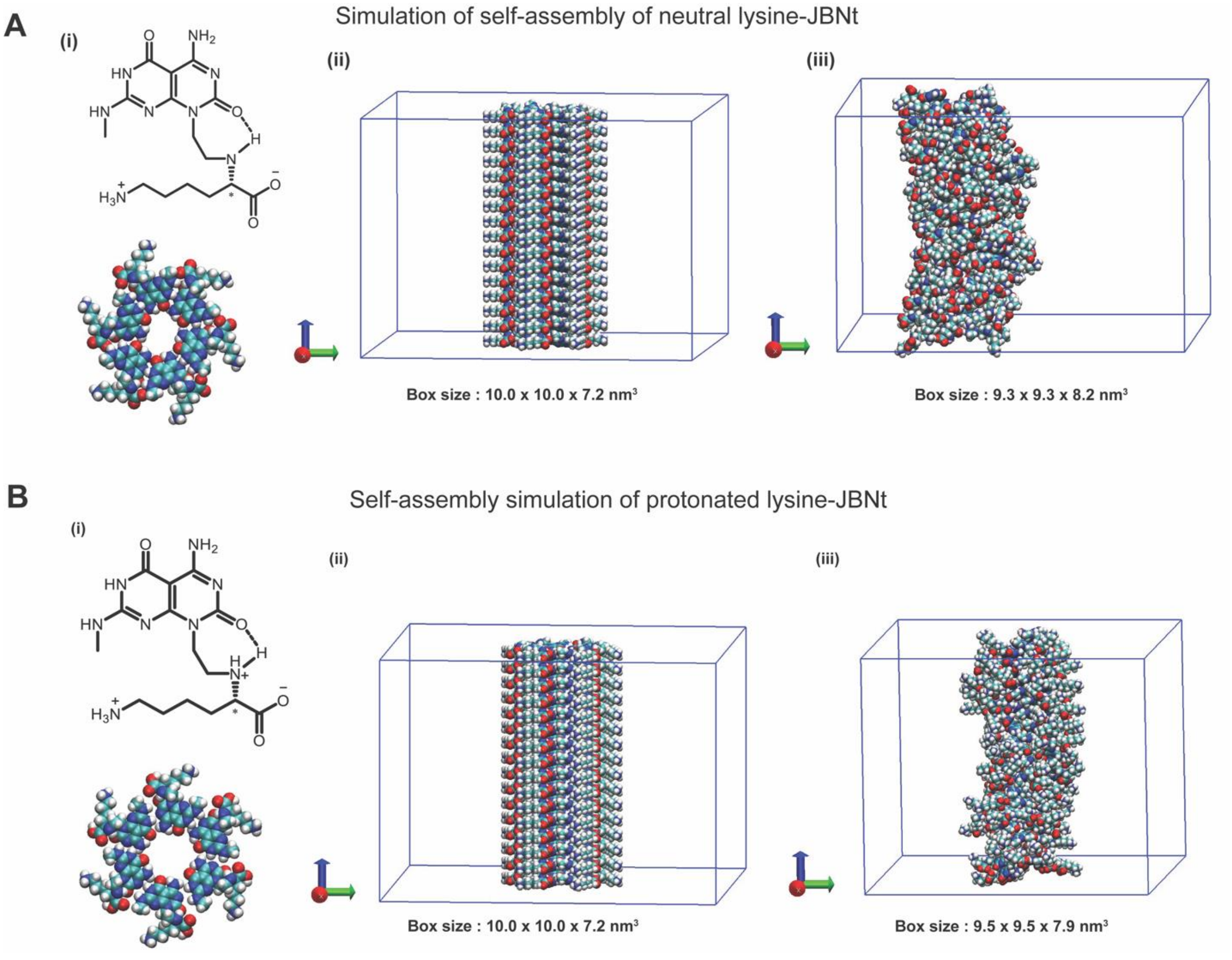

Figure S2. Simulation of self-assembly of Lys-JBNt. (A, i) A ring built form six neutral lysine-based monomers. (A, ii) 16 neutral lysine-based rings to form a JBNt with layer spacing of 4.5 Å. (A, iii) JBNt after 10 ns equilibration MD simulation, before loading DOX simulation. Water molecules are included in the simulation, but not shown here for clarity. (B, i) A ring built from six protonated lysine-based monomers. (B, ii) 16 layers of protonated lysine-based ring to form a JBNt with layer spacing of 4.5 Å. (B, iii) JBNt after 10 ns equilibration MD simulation, before loading DOX simulation. Water molecules and CL^- ions (for neutralization) are included in the simulation, but not shown here for clarity.

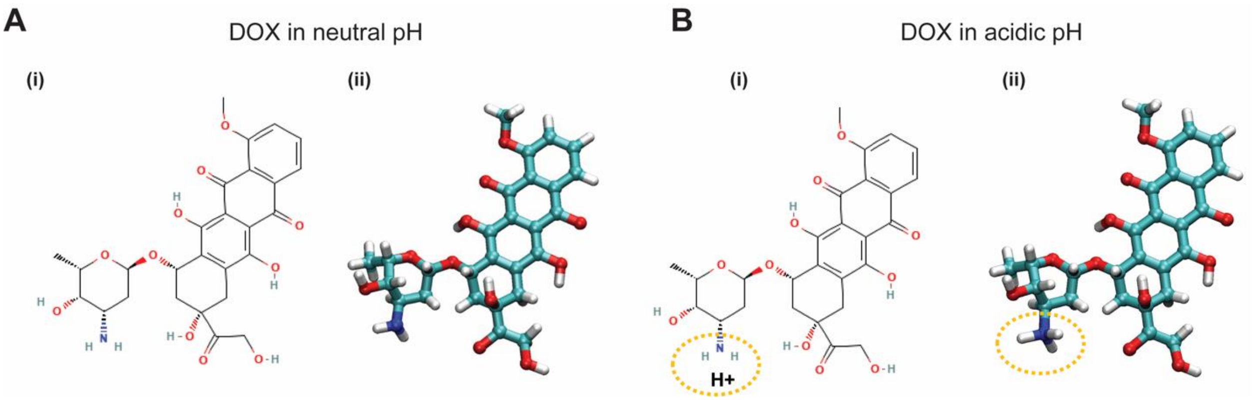

Figure S3. MD simulation of Doxorubicin. (A, i) Chemical structure of DOX in neutral pH. (A, ii) MD simulation of DOX in neutral pH. (B, i) Chemical structure of DOX in acidic pH. (B, ii) MD simulation of DOX in acidic pH.

Histograms of the positions of JBNt throughout the umbrella sampling.

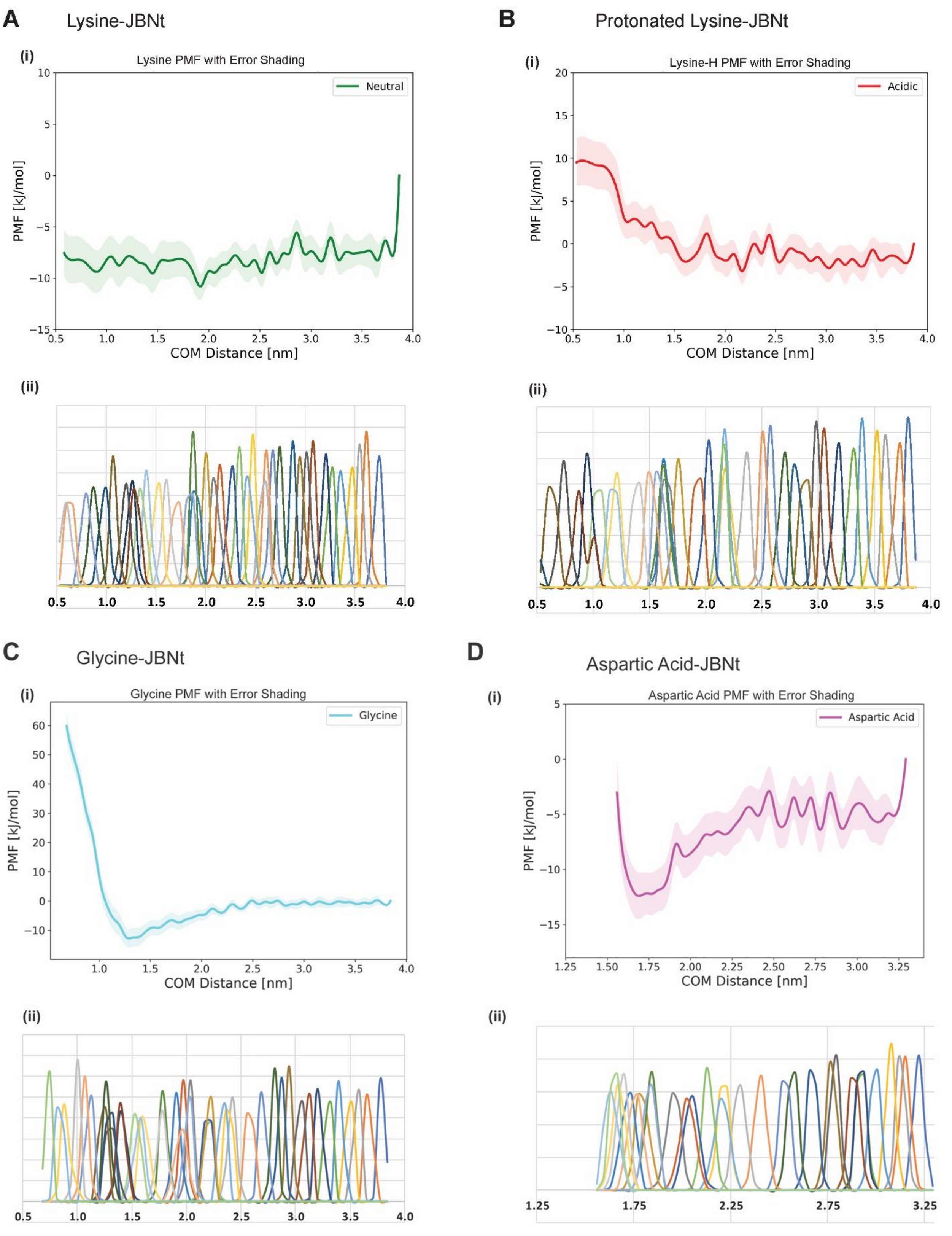

Figure S4. Histogram of the position of JBNt throughout the umbrella sampling. (A, i) Lys-JBNt PMF with error shading. (A, ii) Histogram of Lys-JBNt throughout the umbrella sampling. (B, i) Protonated Lys-JBNt PMF with error shading. (B, ii) Histogram of protonated Lys-JBNt throughout the umbrella sampling. (C, i) Gly-JBNt PMF with error shading. (C, ii) histogram of Gly-JBNt throughout the umbrella sampling. (D, i) Asp-JBNt PMF with error shading. (D, ii) Histogram of Asp-JBNt throughout the umbrella sampling.

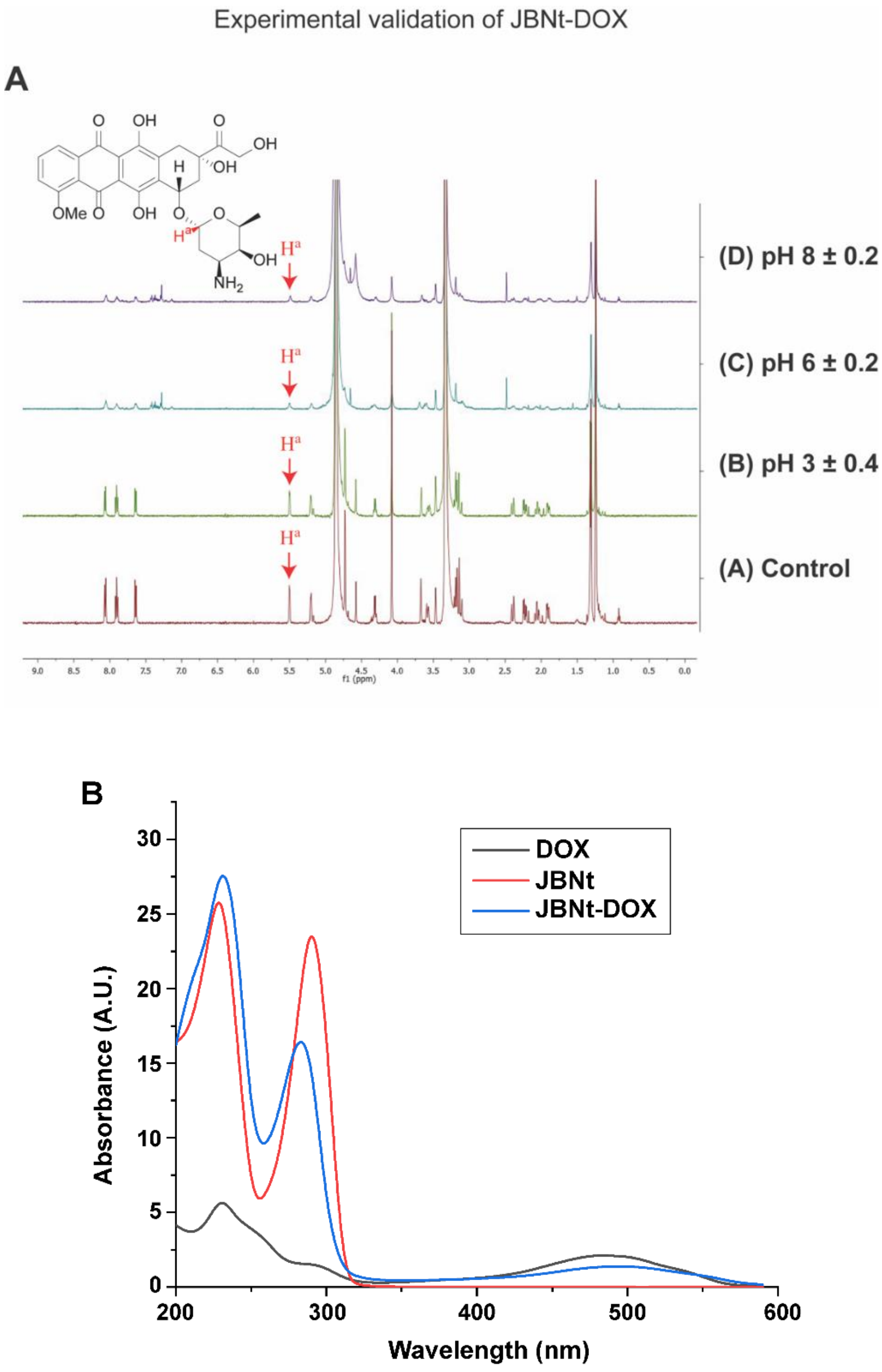

Figure S5. Experimental validation of JBNt-DOX. We assessed the encapsulation efficiencies (EE) across varying pH through nuclear magnetic resonance (NMR). To validate the simulation, the UV-visible (Vis) spectra demonstrated molecular-level incorporation between JBNts and DOX. When assembled with DOX, the 280-nm peak of JBNt-DOX decreased due to the

intercalation between JBNt units and DOX. (A) ^{1}H NMR spectra of control (DOX alone); DOX + JBNp mixtures at different pHs: pH 3±0.4; pH 6±0.2; pH ~8±0.2. (Red arrow points out the Ha peak of DOX demonstrating the loading and unloading of DOX molecules in JBNps) (B) UV-VIS absorbance spectra.

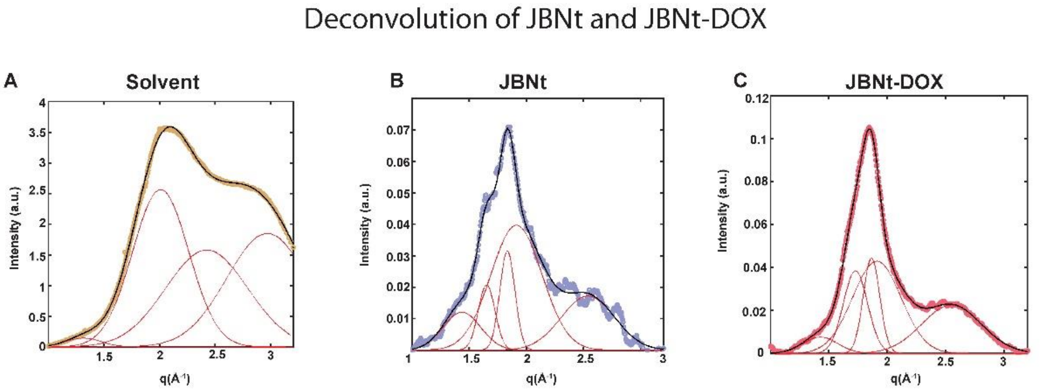

Peak Position (Å⁻¹)	d-Spacing (Å)	Area Contribution (%)
1.32	4.77	1.2
-	-	-
-	-	-

Peak Position (Å⁻¹)	d-Spacing (Å)	Area Contribution (%)
1.44	4.38	10.74
1.65	3.80	8.21
1.83	3.43	11.26

Peak Position (Å⁻¹)	d-Spacing (Å)	Area Contribution (%)
1.43	4.40	4.94
1.73	3.64	17.43
1.87	3.37	13.69

Figure S6. Peak deconvolution analysis of SAXS spectra of JBNt and JBNt-DOX. The peak positions (Å), d-Spacing (Å) and Area contribution (%) are indicated on the tables. (A) Solvent. (B) JBNt. (C) JBNt-DOX.

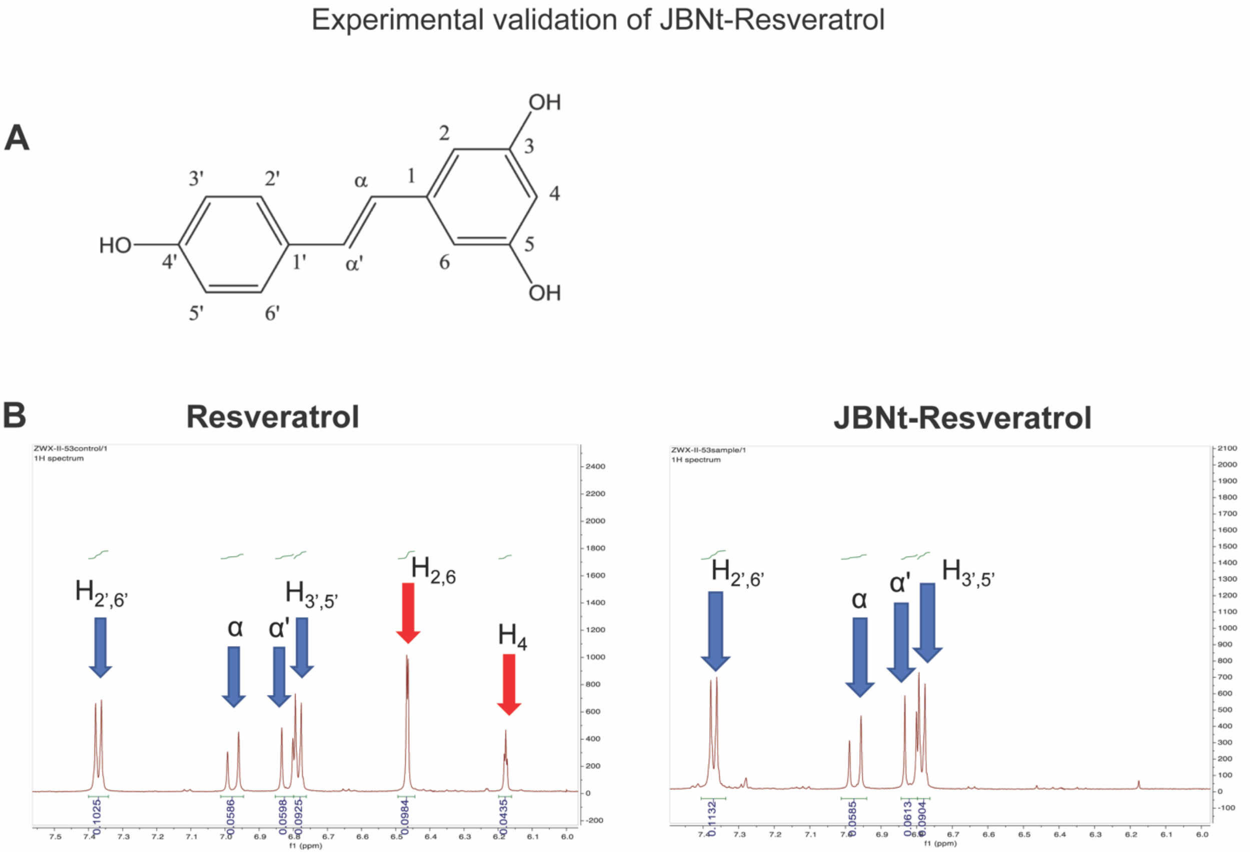

Figure S7. Small molecule (Resveratrol) loaded to JBNt. As an initial demonstration of JBNt ability to incorporate cargoes into JBNt, we also effectively loaded resveratrol, a promising antioxidant and anticancer drug, into JBNt. (A) Chemical structure of Resveratrol. (B) NMR profile of resveratrol and the resveratrol-loaded JBNt (JBNt-Resveratrol, the signals of protons $H_{2,6}$ and H_4 were significantly decreased indicating the dihydroxy benzene ring incorporated into JBNt)

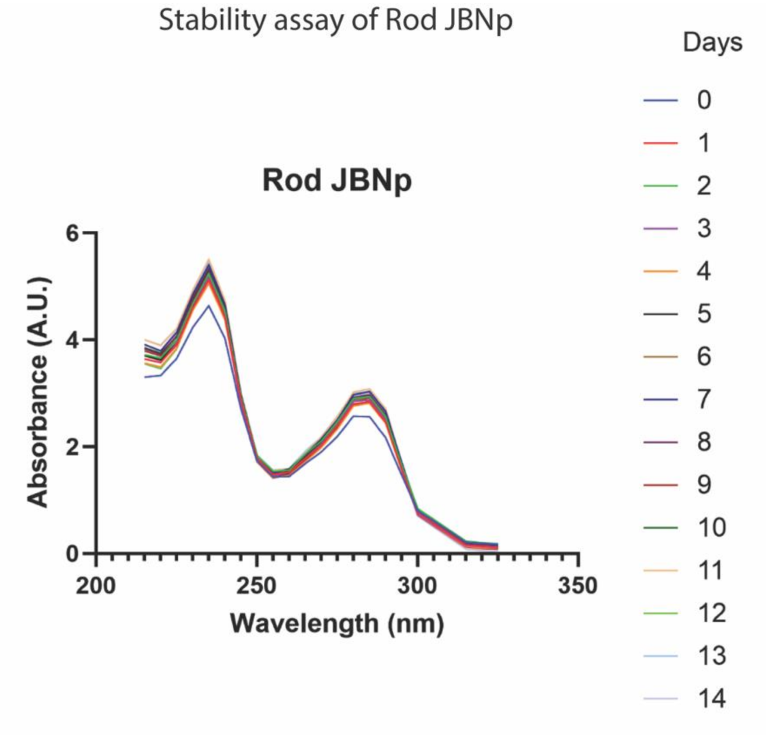

Figure S8. Stability of Rod JBNp. UV-Vis absorbance spectra of DOX-loaded Rod JBNps over a 14-day period demonstrate time-dependent stability. No significant changes were observed in the absorbance spectra, indicating that the JBNps remained stable. These results suggest that Rod JBNps maintain structural integrity over time and can be considered stable.

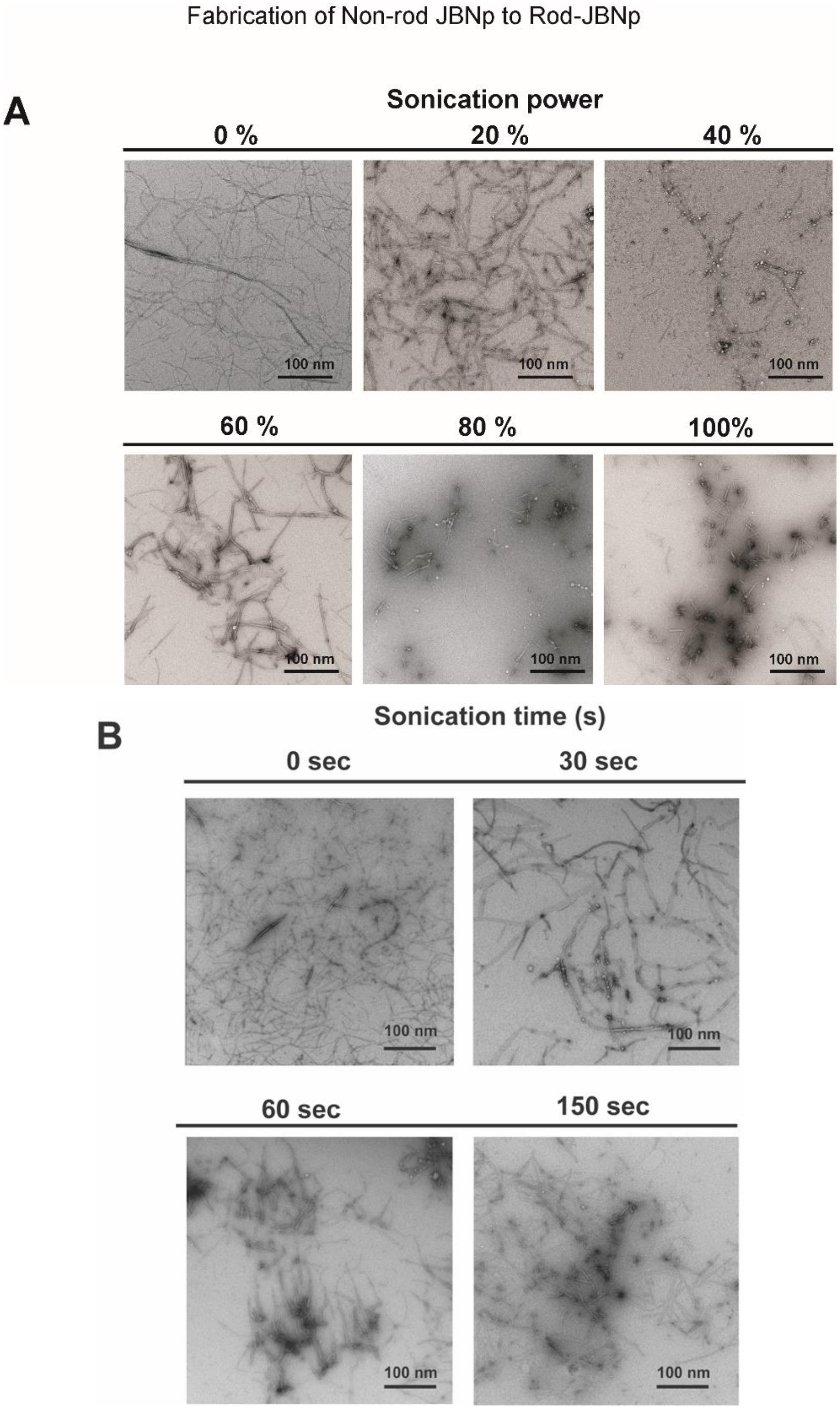

Figure S9. TEM images of JBNp. (A) TEM image showing Non-rod JBNp to Rod JBNp increasing sonication power (sonication time as 150 sec). (B) Rod JBNp varying sonication time (sonication power to 100%)

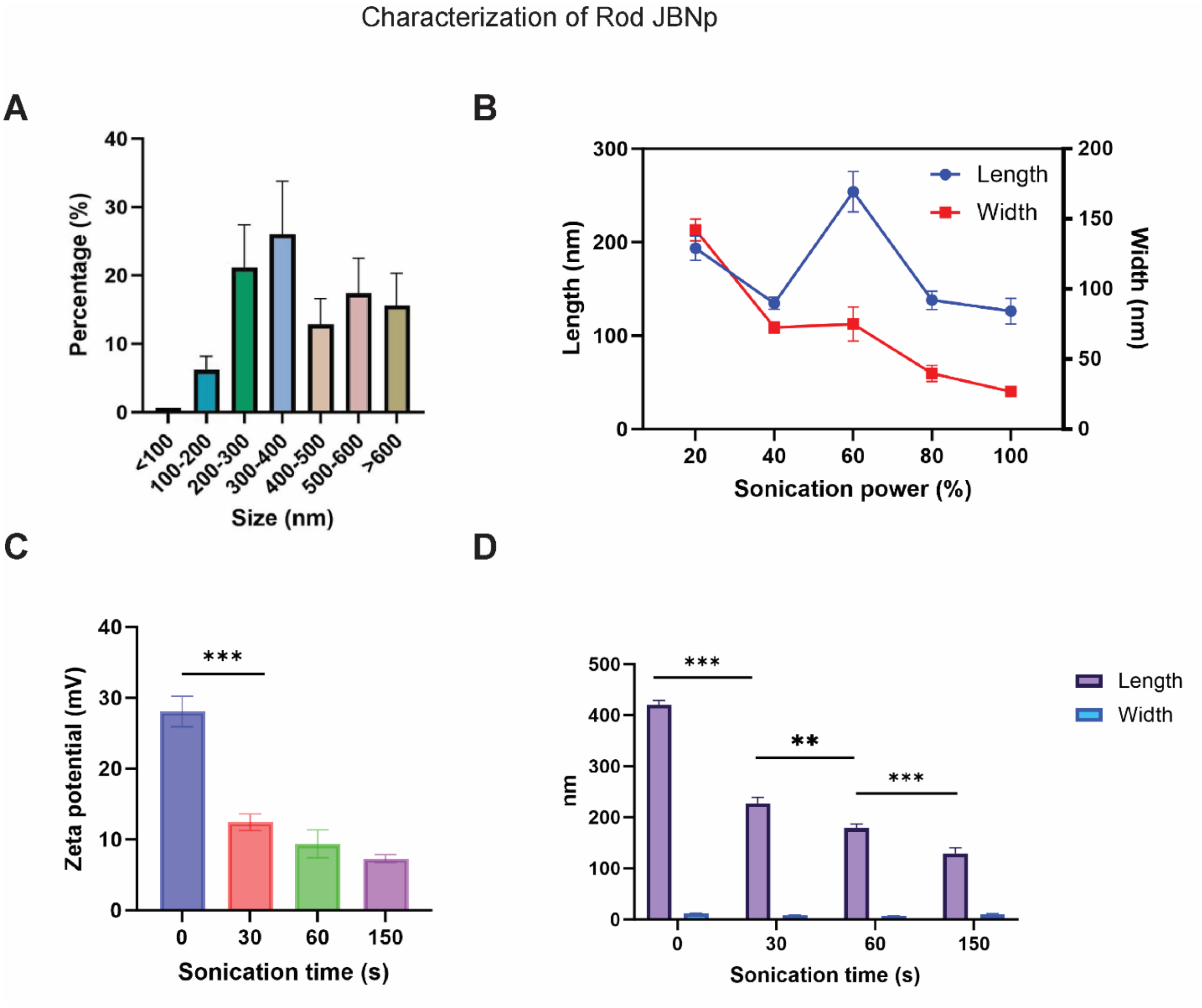

Figure S10. Fabrication and characterization of JBNt-DOX and Rod JBNp. (A) Particle size distribution of the JBNt obtained from the transmission electron microscopy (TEM). (B) Length and width measurement of JBNp varying sonication power (%). (C) Zeta potential of analysis of Rod JBNp varying sonication time (s). (D) Length and width measurement of JBNp varying sonication time (s).

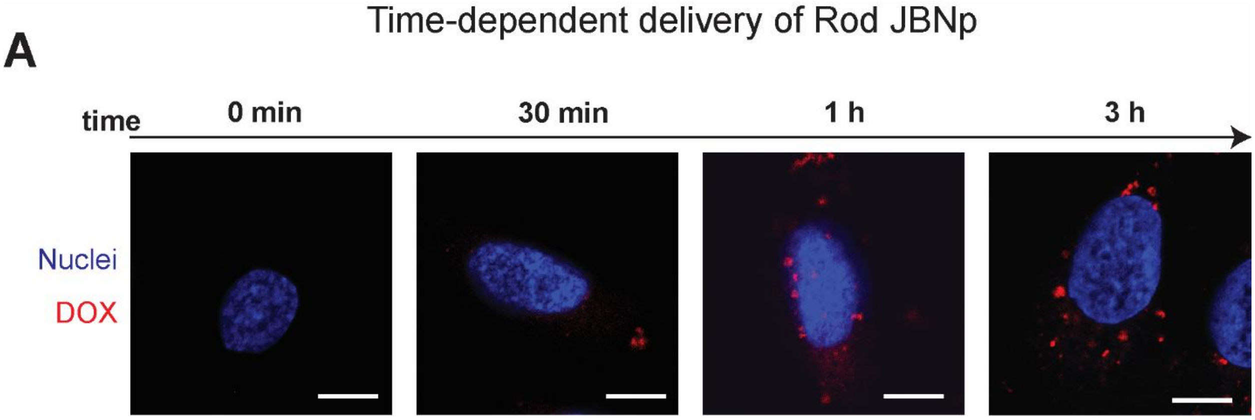

Figure S11. Time-dependent cellular delivery of Rod JBNp. Rod JBNp was able to deliver DOX to cell in a time-dependent fashion, showing red signal (DOX) in cytoplasm (A) Time-dependent delivery of the Rod JBNp to SKOV-3 cells. CLSM images of Rod JBNp delivered DOX (red), cell nuclei (blue). Scale bar = 10 μm.

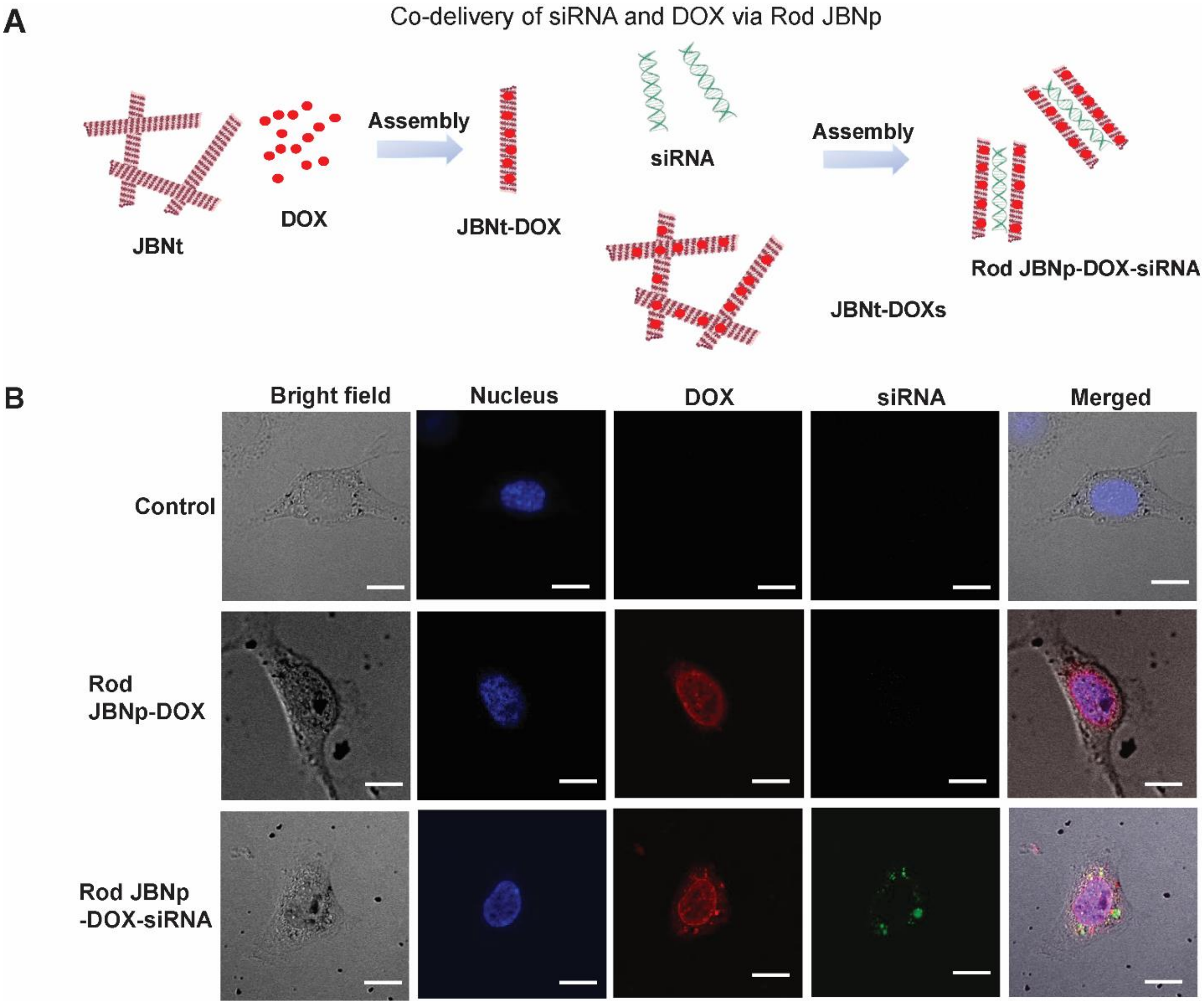

Figure S12. Co-delivery of siRNA and DOX via Rod JBNp in SKOV-3 ovarian cancer cells. As a proof-of-concept, Rod JBNp encapsulate the DOX and siRNA-AF488 (Rod JBNp-DOX-siRNA) and co-deliver them into a 2D cancer cell culture. (A) Schematic drawing of self-assembly of Rod JBNp-DOX-siRNA. (B) CLSM images of co-delivery of DOX (red) and siRNA-AF488 (green) delivered by JBNps; cell nuclei stained with DAPI (blue). Scale bars are 10 μm.

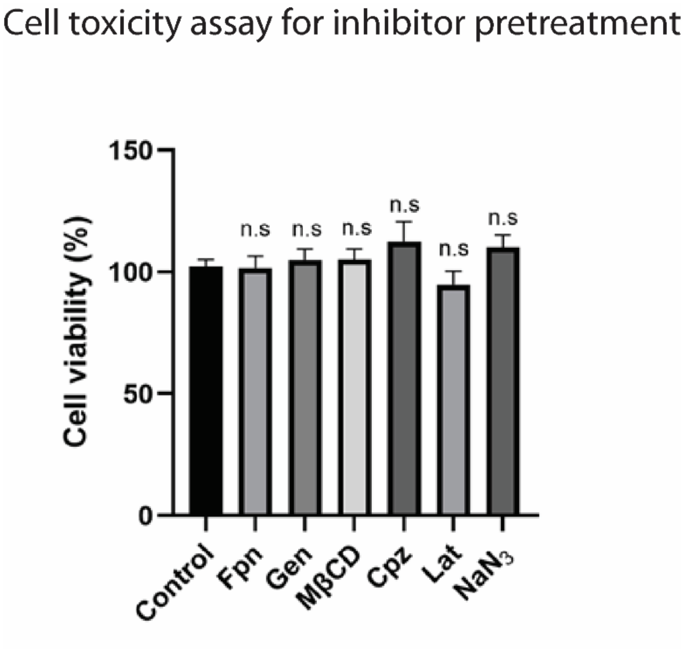

Figure S13. CCK-8 assay for inhibitor-treated cells used in the uptake mechanism study. SKOV-3 cells were exposed to various concentrations of the inhibitors for 30 minutes or 1 hour, which did not induce any significant cytotoxicity. Data are presented as the percentage of viable cells; values represent mean ± SEM ($n \geq 10$). *n.s.* indicates no significant difference ($P > 0.05$) compared to the untreated control.

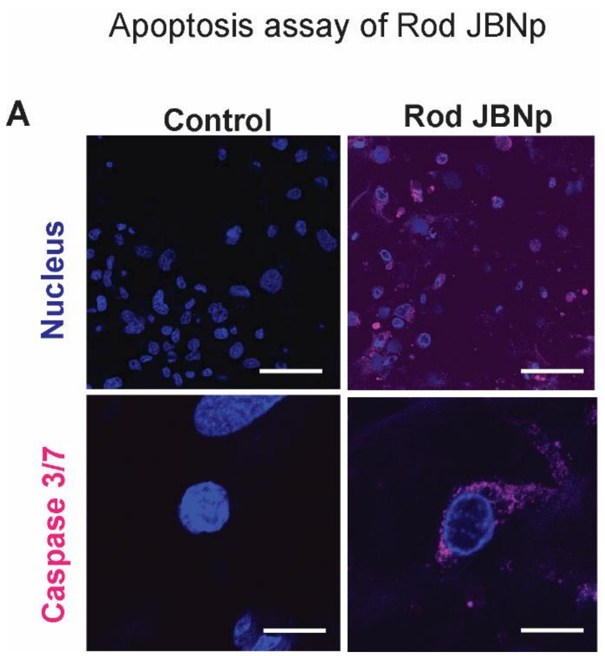

Figure S14. Apoptosis assay of Rod JBNP after staining with Caspase 3/7. Assessment of functional assay with Rod JBNp on SKOV-3 cancer cells and spheroid was performed using Caspase 3/7 staining. Caspase 3/7 activity did significantly increase after 24 h of treatment with Rod JBNp when compared to untreated control in SKOV-3 cells. A) Fluorescence image of Caspase 3/7 reagent stained SKOV-3 human ovarian cells after 24hr transfection of Rod JBNp, or negative control: images taken by CLSM. Scale bars are 20 μm.

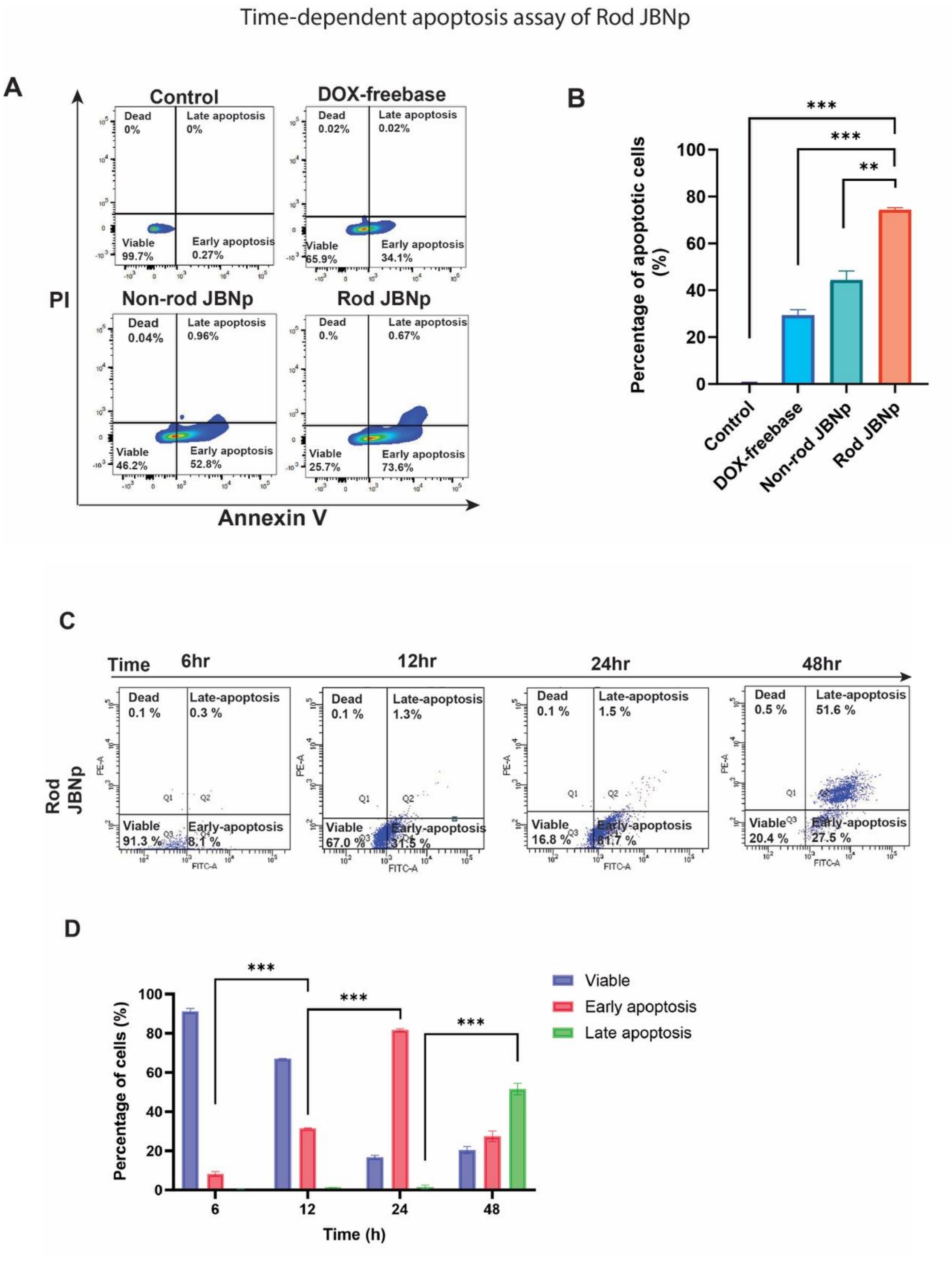

Figure S15. Time-dependent apoptosis assay of Rod JBNp. We demonstrated that the Rod JBNp shows a significantly higher rate of apoptosis by time-dependent manner. (A) Apoptosis assay using flow cytometry after staining with annexin V-FITC/PI. SKOV-3 cells were treated with

indicated groups for 24 h. (B) Percentage of apoptotic cells. n=3. The data were expressed as the percentage of cells, and the values are mean ± SEM ($n \geq 3$). (C) Flow cytometry assay stained with annexin V- FITC/PI. (D) Quantification of apoptosis assay.

Flow-cytometry gating of apoptosis assay

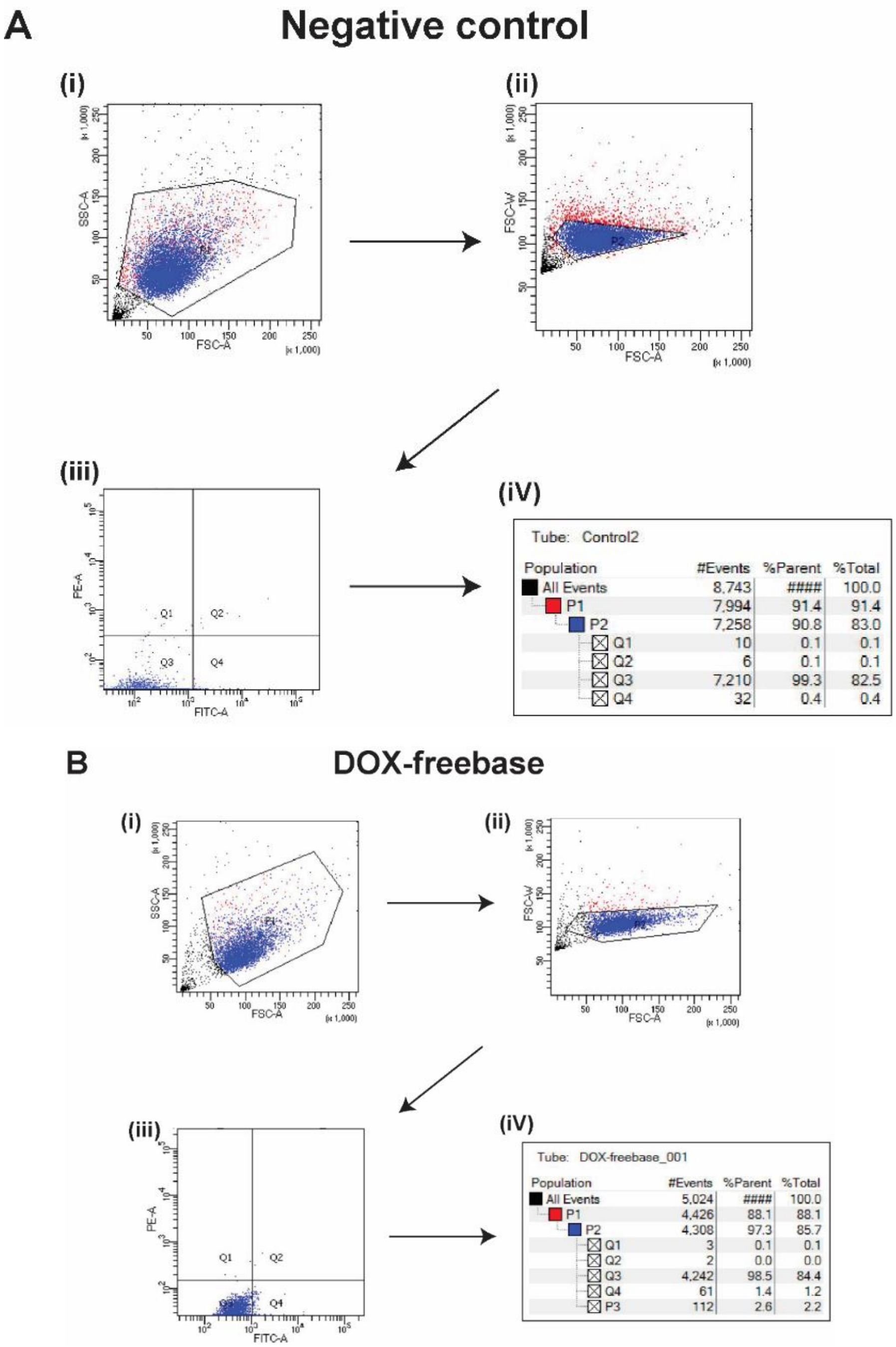

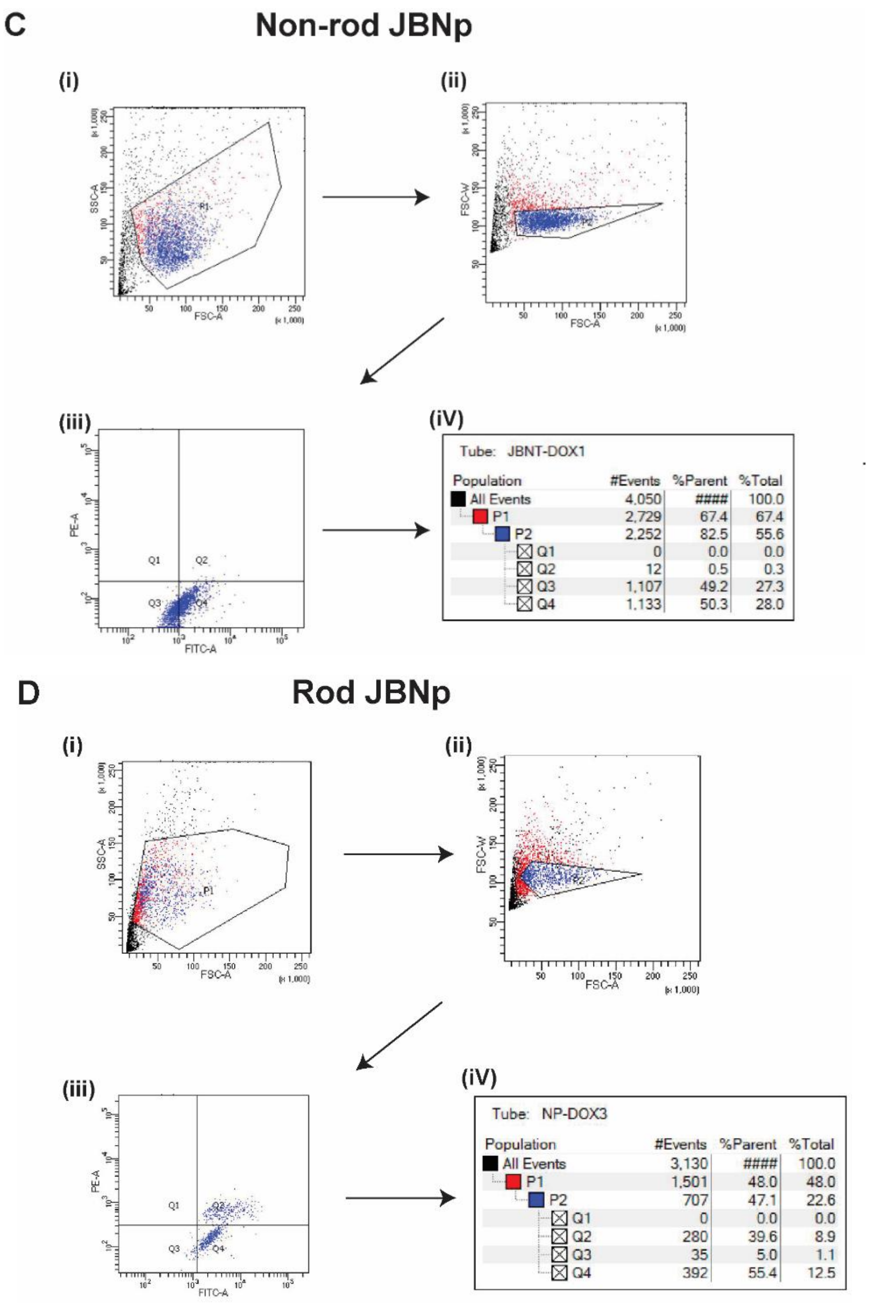

Figure S16. Flow cytometric gating of apoptosis assay. Representative example of gating strategy used to characterize SKOV-3 cells in apoptosis, using co-staining with Annexin-V and propidium iodide (PI). (A) Negative control. (B) DOX-freebase. (C) Non-rod JBNp. (D) Rod JBNp.

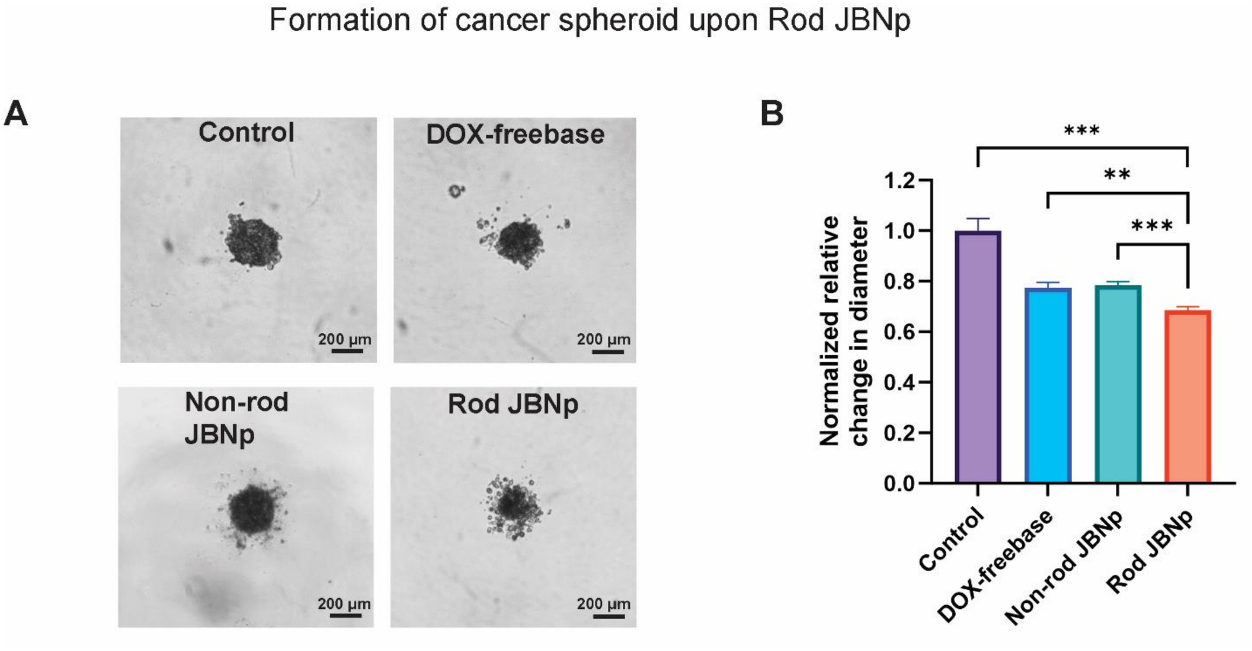

Figure S17. Formation of cancer spheroid upon Rod JBNp. To further test the functional delivery of DOX, we tested the formation of cancer spheroid upon Rod JBNp. Brightfield microscopic images and quantitative spheroid size analysis revealed that Rod JBNp–DOX mediates the initial formation of ovarian carcinoma spheroids better than controls (A) Representative images of formation of SKOV-3 spheroid are mediated by indicative treatment group in 72h (scale bar: 200 μm). (D) Analysis of change in diameter in spheroids. n=3. Data are presented as the means ± SEM of triplicate experiments. *P<0.05, **P<0.01, and ***P<0.001.

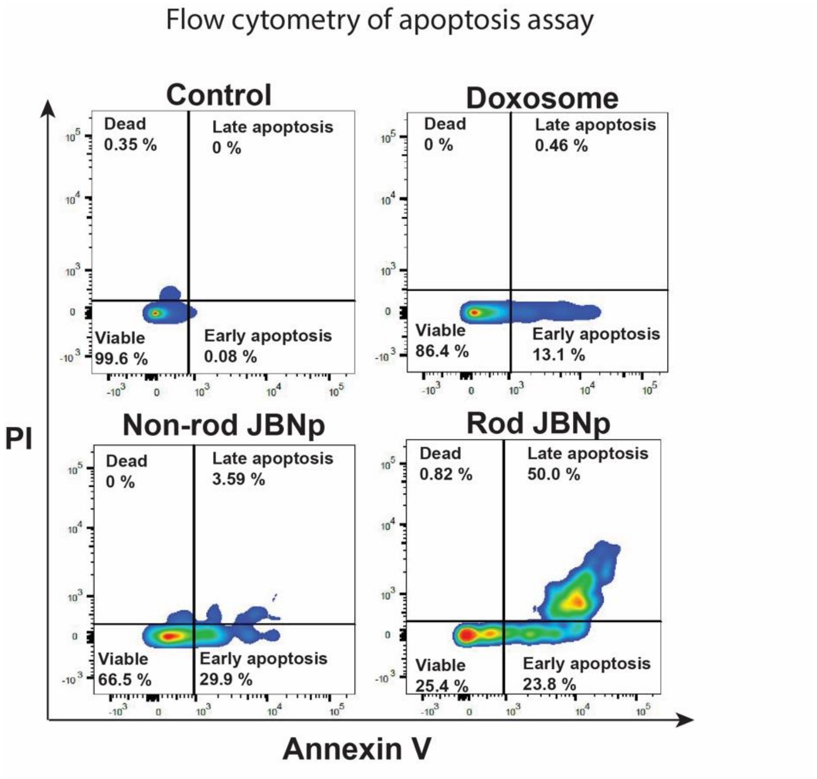

Figure S18. (A) Apoptosis assay of SKOV3 spheroids after treatments for 48h using Apoptosis Kit with Annexin V FITC and PI. Flow cytometry was performed to quantify the Annexin V and PI signals. Percentage of viable, early, and late apoptotic cells. Data are presented as the means ± SEM of triplicate experiments.

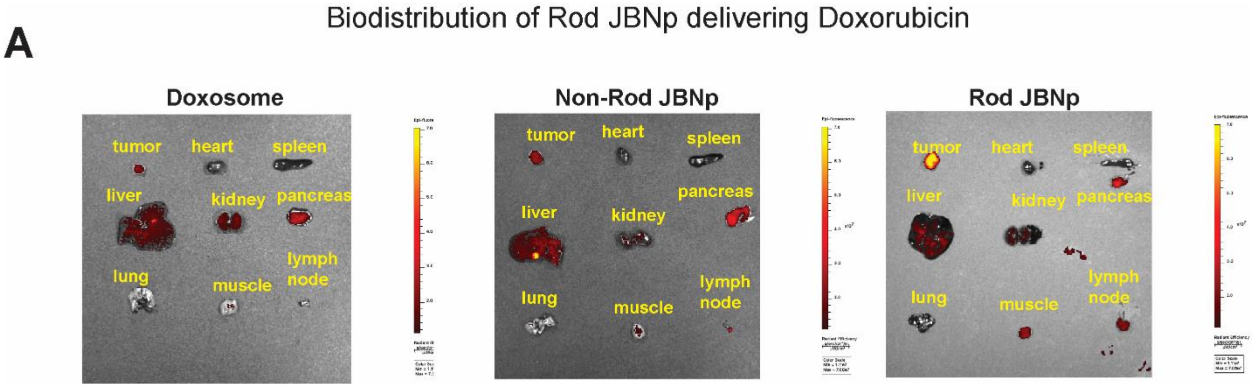

Figure S19. Ex vivo biodistribution of JBNps in mice bearing SKOV-3 tumors. (A) Organs and tumor were removed after 72 h after i.v. injection. In vivo imaging system (IVIS) was used to image tumor, heart, spleen, liver, kidney, pancreas, lung, muscle and lymph node. The color scale to the right indicates radiant efficiency.

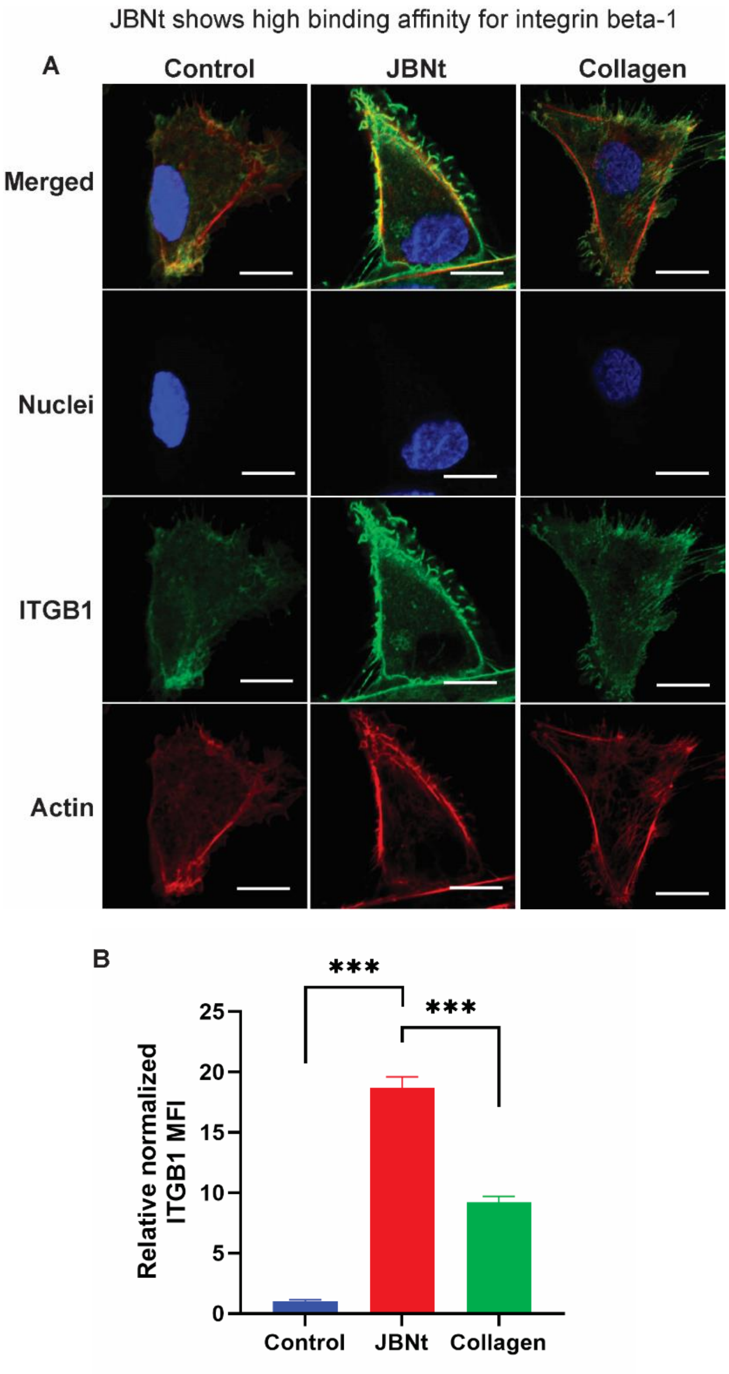

Figure S20. Analysis of Integrin beta-1 (ITGB1) binding to the JBNt. JBNt shows high binding affinity for ITGB1 (A) Analysis of immunofluorescence staining of ITGB1 in SKOV3 cells. JBNts were pre-coated (concentration of 0.6 μg/mm^2) to silicone slide and seeded with SKOV3 for 24hr. JBNt coated group shows higher ITGB1 (green) fluorescence intensity than collagen coated (positive control) and without coated (negative control) group. Scale bar : 10 μm. (B)

Quantification of normalized mean fluorescence intensity (MFI). The values are mean ± SEM ($n \geq 10$). *$p < 0.05$, ** $p < 0.01$, and *** $p < 0.001$ compared to control.

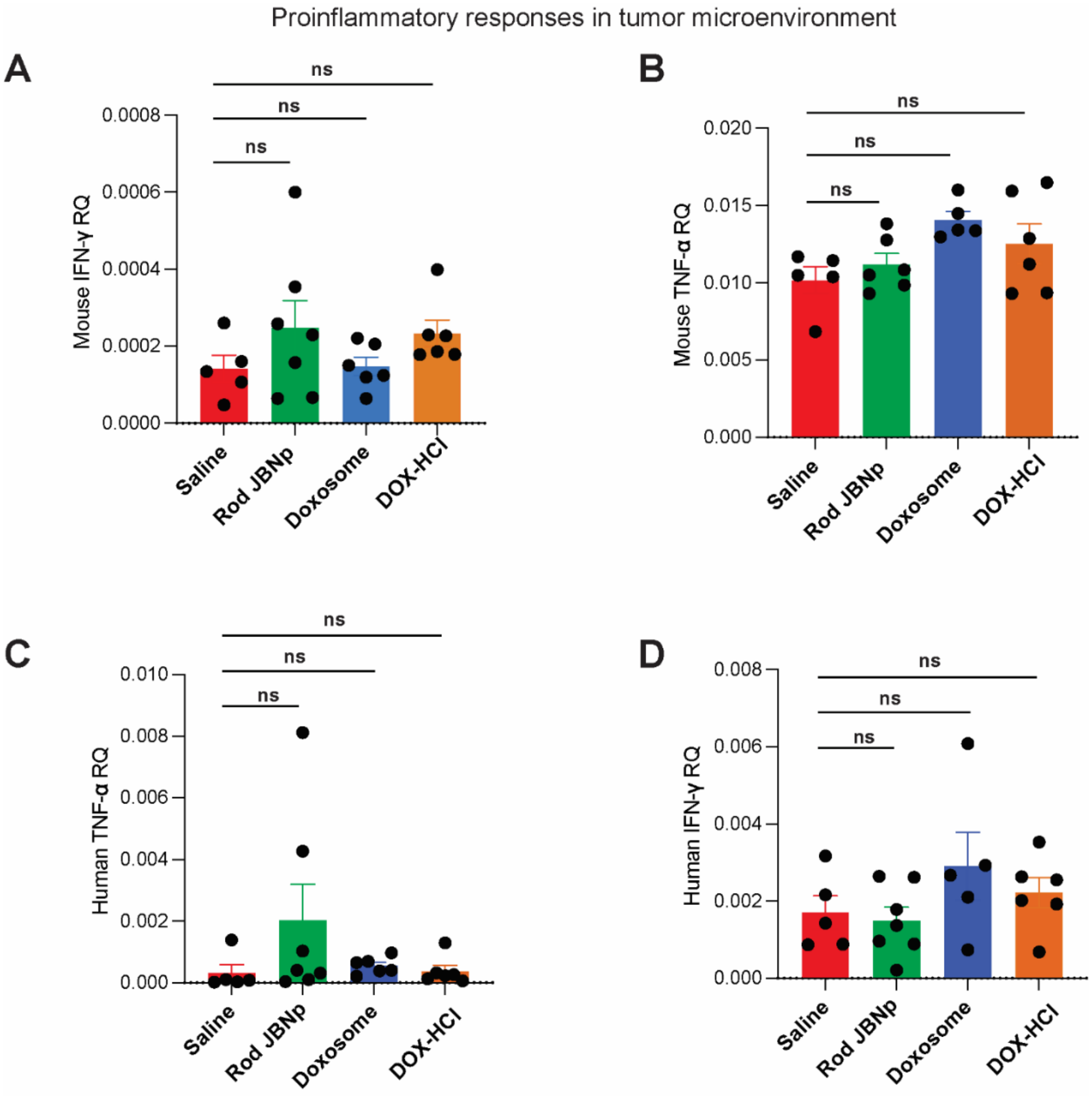

Figure S21. Quantification of proinflammatory cytokines mouse and human IFN-γ mRNA and TNF-α mRNA in tumor. To exclude the inflammation effect in apoptosis, we have also confirmed that the proinflammatory responses in tumor microenvironment are not playing a role in therapy response. (A) Quantitative PCR (qPCR) was conducted to examine the Mouse IFN-γ mRNA levels in tumor tissues. (B) Mouse TNF-α mRNA. (C) human IFN-γ mRNA. (D) human TNF-α mRNA. GAPDH mRNA for human and mouse were used as housekeeping control, respectively. The data presented as the mean ± SEM assessed by the One-way ANOVA test.

Supplementary Tables

Coded Coefficients					
**Term**	**Coefficient**	**SE Coef**	**T-Value**	**P-Value**	**VIF**
Constant	0.4008	0.0414	9.67	0	
pH	0.2827	0.0183	15.46	0	1.2
Temp (C)	0.026	0.0184	1.42	0.166	1.19
Time (h)	0.0557	0.0192	2.89	0.006	1.03
pH*pH	-0.0185	0.0338	-0.55	0.587	1.04
Temp (C)*Temp (C)	-0.1019	0.0315	-3.24	0.003	1.02
Time (h)*Time (h)	-0.0516	0.0338	-1.52	0.136	1
pH*Temp (C)	0.0342	0.0203	1.69	0.1	1.03
pH*Time (h)	0.0659	0.0228	2.88	0.007	1.17
Temp (C)*Time (h)	0.0146	0.0231	0.63	0.53	1.16

Table S1. Coded regression coefficients for the RSM model assessing the effects of pH, temperature, and time on encapsulation efficiency (EE%). This table includes the estimated coefficients, standard errors, t-values, p-values, and variance inflation factors (VIF) for linear, quadratic, and interaction terms in the coded regression model. Regression equation derived from the RSM model, presented in both coded and uncoded units. The equation describes the relationship between EE% and the input variables (pH, temperature, and time), including quadratic and interaction terms, to guide optimization within the experimental design space.

EE (%) = '-0.318 + 0.0928 pH + 0.01130 Temp (C) + 0.00011 Time (h) - 0.00254 pH*pH - 0.000353 Temp (C)*Temp (C) - 0.000023 Time (h)*Time (h) + 0.000746 pH*Temp (C) + 0.000511 pH*Time (h) + 0.000018 Temp (C)*Time (h)

Let pH = x, Temp (C) = y, Time (h) = z

EE (%) =

$$'-0.318+0.0928x+0.01130y+0.00011z-0.00254x^2-0.000353y^2-0.000023z^2+0.000746xy+0.000511xz+0.000018yz$$

Multiple Response Prediction		
**Variable**	**Optimal Value**	
pH	8.3	
Temp (C)	27.3535	
Time (h)	96	
**Response**	**Fit**	**SE Fit**
EE (%)	0.7488	0.0492
**95% Confidence Interval**		
(0.6489, 0.8488)		
**95% Prediction Interval**		
(0.5348, 0.9628)		

Table S2. Summary of RSM model performance metrics and optimal conditions for maximizing EE%. Includes predicted response value with standard error, confidence intervals, and prediction intervals at the optimal settings of pH = 8.3, temperature = 27.35°C, and time = 96 h.

Model Summary			
**S**	**R-sq**	**R-sq(adj)**	**R-sq(pred)**
0.0932141	89.49%	86.78%	82.30%

Analysis of Variance					
**Source**	**DF**	**Adj SS**	**Adj MS**	**F-Value**	**P-Value**
Model	9	2.58875	0.28764	33.1	0
Linear	3	2.20791	0.73597	84.7	0
pH	1	2.07761	2.07761	239.11	0
Temp (C)	1	0.01742	0.01742	2.01	0.166
Time (h)	1	0.0728	0.0728	8.38	0.006
Square	3	0.11385	0.03795	4.37	0.01
pH*pH	1	0.00261	0.00261	0.3	0.587
Temp (C)*Temp (C)	1	0.09105	0.09105	10.48	0.003
Time (h)*Time (h)	1	0.0202	0.0202	2.32	0.136
2-Way Interaction	3	0.10054	0.03351	3.86	0.017
pH*Temp (C)	1	0.02476	0.02476	2.85	0.1
pH*Time (h)	1	0.07228	0.07228	8.32	0.007
Temp (C)*Time (h)	1	0.0035	0.0035	0.4	0.53
Error	35	0.30411	0.00869		
Total	44	2.89286			

Table S3. Model summary statistics for RSM including residual standard deviation (S), R-squared values, and analysis of variance (ANOVA).The table summarizes key statistical indicators of model quality, including goodness-of-fit (R^2), adjusted R^2, predicted R^2, and residual standard deviation, along with detailed ANOVA results for individual model terms. The standard deviation of the model (S = 0.0932) is significantly lower than that of the experimental data (SD = 0.2564), indicating improved consistency and predictive accuracy. S = standard deviation of the residuals (standard error of the regression).

Drug loading rates calculation.

pH	<3.6	~6.5	~7.5	~8.5
EE%[1]	0	45	88	93
LC%[2]	0	6.6	13	14

Table S4. Doxorubicin (DOX) loading rates at different pHs. [1]Encapsulation efficiency: EE% = $\frac{DOX_{total}-DOX_{free}}{DOX_{total}}$; [2]Loading capacity: LC% = $\frac{DOX_{total}-DOX_{free}}{JBNTs}$.

Complete blood count (CBC) analysis

DOX-HCl

Parameter	Base line	After treatment
**Red Blood Cell (10^{12} cells / liter)**	10.63 $\pm$ 0.20	7.31 $\pm$ 0.73
**White Blood Cell (10^{9} cells / liter)**	4.81 $\pm$ 1.24	5.80 $\pm$ 1.45
**Platelet (10^{9} cells / liter)**	580 $\pm$ 4.58	1047 $\pm$ 83.26
**Hemoglobin (g/l)**	14.57 $\pm$ 0.23	11.9 $\pm$ 0.96
**Hematocrit (%)**	47.0 $\pm$ 0.87	36.14 $\pm$ 3.37
**Neutrophil (10^{9} cells / liter)**	1.29 $\pm$ 0.46	3.79 $\pm$ 1.03
**Lymphocyte (10^{9} cells / liter)**	3.23 $\pm$ 0.83	1.38 $\pm$ 0.27
**Monocyte (10^{9} cells / liter)**	0.29 $\pm$ 0.08	0.63 $\pm$ 0.27
**Mean corpuscular volume (fL)**	44.3 $\pm$ 0.67	49.67 $\pm$ 2.19

Doxosome

Parameter	Base line	After treatment
**Red Blood Cell (10^{12} cells / liter)**	10.04 $\pm$ 0.71	8.45 $\pm$ 0.14
**White Blood Cell (10^{9} cells / liter)**	5.04 $\pm$ 0.96	5.96 $\pm$ 0.66
**Platelet (10^{9} cells / liter)**	609.2 $\pm$ 40.3	853 $\pm$ 38.3
**Hemoglobin (g/l)**	13.8 $\pm$ 0.78	12.12 $\pm$ 0.19
**Hematocrit (%)**	44.65 $\pm$ 2.49	39.86 $\pm$ 0.77
**Neutrophil (10^{9} cells / liter)**	1.58 $\pm$ 0.21	3.70 $\pm$ 0.44
**Lymphocyte (10^{9} cells / liter)**	3.02 $\pm$ 0.76	1.70 $\pm$ 0.74
**Monocyte (10^{9} cells / liter)**	0.43 $\pm$ 0.04	0.55 $\pm$ 0.73
**Mean corpuscular volume (fL)**	44.8 $\pm$ 1.16	47.4 $\pm$ 0.68

Rod JBNp

Parameter	Base line	After treatment
**Red Blood Cell (10^{12} cells / liter)**	9.72 ± 0.95	8.38 ± 0.51
**White Blood Cell (10^9 cells / liter)**	3.12 ± 0.37	3.98 ± 0.32
**Platelet (10^9 cells / liter)**	700 ± 67.03	797.25 ± 52.63
**Hemoglobin (g/l)**	13.43 ± 1.42	11.68 ± 1.04
**Hematocrit (%)**	42.98 ± 3.66	39.12 ± 2.99
**Neutrophil (10^9 cells / liter)**	1.32 ± 0.09	1.99 ± 0.39
**Lymphocyte (10^9 cells / liter)**	1.52 ± 0.36	1.70 ± 0.68
**Monocyte (10^9 cells / liter)**	0.27 ± 0.04	0.28 ± 0.07
**Mean corpuscular volume (fL)**	44.5 ± 0.87	46.5 ± 0.87

Table S5. Result of the complete blood count (CBC) study. **(A)** CBC values of DOX-HCl, Doxosome and Rod JBNp including Red blood cells (RBC), White blood cells (WBC), Platelets (PLT), Hemoglobin (HGB), Hematocrit (HCT), Neutrophil (NEU), Lymphocyte (LYM), Monocyte (MON), and Mean corpuscular volume (MCV) expressed as mean and standard error of mean (SEM), evaluated in SKOV-3 tumor bearing mice group composed of 5 Nu/J mice.
